# L-Phenylalanine promotes liver steatosis by inhibiting BNIP3-mediated mitophagy

**DOI:** 10.1186/s10020-025-01303-5

**Published:** 2025-06-30

**Authors:** Ying Sun, Lingli Cai, Bowei Yu, Haojie Zhang, Ziteng Zhang, Xiaoqin Xu, Yuefeng Yu, Jiang Li, Chi Chen, Fangzhen Xia, Yingli Lu, Kun Zhang, Ningjian Wang

**Affiliations:** 1 Institute and Department of Endocrinology and Metabolism, Shanghai Ninth People’s Hospital, Shanghai JiaoTong University School of Medicine, Shanghai, 200011 China; 2 Department of Endocrinology and Metabolism and Department of Guideline and Rapid Recommendation, Cochrane China Centre, MAGIC China Centre, Chinese Evidence-Based Medicine Centre, West China Hospital, Sichuan University, Chengdu, China; 3 Department of Endocrinology, Shuguang Hospital Affiliated to Shanghai University of Traditional Chinese Medicine, Shanghai, China

**Keywords:** Liver steatosis, L-Phenylalanine, Mitophagy, BNIP3

## Abstract

**Background:**

L-Phenylalanine (L-Phe) levels are elevated in patients with metabolic dysfunction-associated steatotic liver disease (MASLD). However, whether L-Phe induces liver steatosis and the underlying mechanism remain unknown. This study aimed to investigate the mechanism through which L-Phe promotes liver steatosis.

**Methods:**

We utilized human data from the UK Biobank and SPECT-China studies. Plasma/serum samples were collected for metabolomic testing to measure L-Phe levels. A rat model with L-Phe in the drinking water was established to investigate changes in hepatic lipid metabolism. In addition, BNIP3 was overexpressed both in vitro and in vivo to validate the role of L-Phe in BNIP3-mediated mitophagy associated with liver steatosis.

**Results:**

In both populations, elevated L-Phe quartiles were associated with increased body mass index, triglyceride, and transaminase levels and increased odds of MASLD (all *p* < 0.05). Rats exposed to L-Phe had increased hepatic lipid deposition and decreased mitophagy in the liver. Differentially expressed proteins were enriched in the PPARα and fatty acid β-oxidation signalling pathways, with downregulation of the mitophagy marker BNIP3. Mitophagy was activated by rapamycin and then inhibited by L-Phe, indicating that elevated L-Phe promoted lipid accumulation by suppressing mitophagy. BNIP3 overexpression effectively mitigated L-Phe-induced hepatic steatosis by restoring mitophagy. Moreover, L-Phe regulates the BNIP3-mediated PPARα and AMPK/mTOR signalling pathways to promote hepatic steatosis.

**Conclusions:**

Our study revealed the role of L-Phe in regulating lipid metabolism and promoting liver steatosis via BNIP3-mediated mitophagy. These findings provide novel insights into the link between L-Phe and liver steatosis, suggesting potential nutritional intervention strategies for preventing MASLD.

**Supplementary Information:**

The online version contains supplementary material available at 10.1186/s10020-025-01303-5.

## Introduction

Non-alcoholic fatty liver disease, recently renamed metabolic dysfunction-associated steatotic liver disease (MASLD), has emerged as a prevalent chronic condition that will affect one-third of the population worldwide by 2030 (Powell et al. 2021; Estes et al. 2018). It is a leading cause of non-alcoholic steatohepatitis, hepatocellular carcinoma, and end-stage liver disease, imposing substantial burdens on individuals and healthcare systems (Younossi et al. 2018). Given the absence of effective pharmacological therapies for MASLD, early identification of risk factors and reasonable nutritional intervention are warranted (Younossi et al. 2018). Excessive deposition of fat in the liver, known as liver steatosis, is a hallmark of MASLD. Recent studies have focused on revealing the potential mechanisms of liver steatosis to advance primary prevention strategies and discover therapeutic targets for MASLD (Ahn 2023).

Circulating amino acids are important for fundamental physiological functions, including protein synthesis and energy and lipid metabolism. Emerging clinical studies have provided insights into the close relationship of amino acids with steatohepatitis and MASLD development (Kalhan et al. 2011; Lake et al. 2015; Gaggini et al. 2018). However, evidence linking specific amino acids to MASLD or related liver steatosis remains limited, particularly when large populations are integrated with experimental findings to explore the underlying mechanisms involved. L-phenylalanine (L-Phe), an essential amino acid, is frequently altered in individuals with metabolic conditions such as type 2 diabetes, obesity, and insulin resistance, all of which are components of MASLD (Adeva et al. 2012; Luo et al. 2020; Wu et al. 2021). Several studies have reported elevated levels of L-Phe in the plasma metabolome of patients with MASLD (Kalhan et al. 2011; Lake et al. 2015; Julkunen et al. 2023). Our previous research identified high consumption of artificially sweetened beverages as an independent risk factor for MASLD, with L-Phe being a major metabolite of aspartame, one of the main artificial sweeteners in these beverages (Sun et al. 2023a). However, whether increased levels of L-Phe contribute to the dysregulation of hepatic lipid metabolism, thereby leading to MASLD, remains unclear. Moreover, the normal range of amino acid levels in the body varies among different populations or species, especially regarding essential amino acids obtained through dietary intake (Gaggini et al. 2018; Doestzada et al. 2022; Yeung et al. 2022). It is still uncertain whether elevated levels of L-Phe pose potential risks for liver steatosis.

MASLD manifests from the abnormal accumulation of fat in hepatocytes, which is driven by increased fatty acid synthesis or decreased lipolysis, both of which are processes intricately involving mitochondria. Mitochondrial structural and functional impairments lead to reduced mitochondrial β-oxidation of free fatty acids and lipid peroxidation (Ciaula et al. 2021). At this time, selective autophagy, namely, mitophagy, plays a pivotal role in eliminating damaged or dysfunctional mitochondria to maintain normal lipid metabolism (Ma et al. 2020). Previous studies have revealed that BNIP3 can activate mitophagy through the atypical pathway, concurrently influencing signalling cascades to regulate hepatic lipid metabolism (Chen et al. 2024; Springer et al. 2021). Moreover, emerging evidence from in vitro and in vivo studies suggests a potential role for circulating L-Phe in modulating hepatic autophagy processes (Seglen et al. 1980; Mortimore et al. 1983). However, whether there was a role of L-Phe in mitophagy in liver still unclear. Hence, exploration is warranted to elucidate whether L-Phe may induce liver steatosis via its effects on mitophagy.

In this study, we initially observed a significant positive association between L-Phe levels and hepatic lipid metabolism and the prevalence of MASLD in two different populations. Subsequently, by providing L-Phe-containing water to rats, we confirmed liver steatosis and decreased mitophagy. We further conducted rescue experiments by overexpressing BNIP3, a key protein involved in mitophagy, both in vivo and in vitro to evaluate liver steatosis and mitophagy. These findings provide novel insights into the potential role of L-Phe in liver steatosis via the inhibition of BNIP3-mediated mitophagy.

## Materials and methods

### Participants

In the UK Biobank, a nationwide cohort study, participants completed touch screen questionnaires, physical measurements, face-to-face interviews, and biological sample collection (Sudlow et al. 2015). A total of 183,842 participants free of any diagnosed liver disease, without excessive alcohol consumption (men ≥ 30 g/day, women ≥ 20 g/day), and with complete metabolomics data were included in the analyses (Figure S1). The UK Biobank received ethical approval from the North West Multicentre Research Ethics Committee (REC reference 11/NW/0382).

SPECT-China (Survey on the Prevalence in East China for Metabolic Diseases and Risk Factors) was established to explore risk factors for metabolic diseases in Chinese adults. Detailed information regarding the recruitment for SPECT-China has been previously described (Sun et al. 2024). A total of 155 participants without acute illness or severe communication impairments, without excessive alcohol consumption or a history of hepatitis, and who underwent abdominal ultrasound examinations with available metabolomics data were eligible for the analyses (Figure S1). The SPECT-China study was approved by the Ethics Committee of Shanghai Ninth People’s Hospital, Shanghai JiaoTong University School of Medicine (ChiCTR1800017573).

All research was conducted in accordance with both the Declaration of Helsinki and the Declaration of Istanbul. Written consent was given by all subjects.

### Biochemical metabolomics evaluation in humans

In the UK Biobank, EDTA plasma samples were prepared by the UK Biobank Laboratory (Stockport, UK), shipped to Nightingale Health Laboratories in Finland on dry ice and then stored in a freezer at − 80 °C. Details of the metabolic biomarker profiling platform and experimentation have been described previously (Julkunen et al. 2023); briefly, 249 metabolic biomarkers were measured from randomly selected EDTA plasma samples using high-throughput nuclear magnetic resonance spectroscopy (NMR). The measurements were taken between June 2019 and April 2020 (Phase 1) and between April 2020 and June 2022 (Phase 2) using eight spectrometers (Bruker AVANCE IIIHD) from approximately 280,000 UK Biobank participants. The average biomarker detection rate was > 99% across the plasma samples, and the biomarkers were quantified using Nightingale Health’s proprietary software (quantification library 2020). The coefficients of variation for each biomarker are given in the UK Biobank data resource (https://biobank.ndph.ox.ac.uk/showcase/label.cgi?id=220).

In SPECT-China, venous serum samples were obtained after fasting for at least 8 h, immediately centrifuged, and stored at − 20 °C. All plasma and serum samples were transferred to the central laboratory on dry ice within 2 to 4 h and stored at − 80 °C. Biochemical indices, including triglyceride (TG), total cholesterol (TC), high-density lipoprotein cholesterol (HDL-C), low-density lipoprotein cholesterol (LDL-C), alanine aminotransferase (ALT) and aspartate aminotransferase (AST) levels, were analysed with a Beckman Coulter AU 680 analyser (Beckman Coulter, Brea, CA). Untargeted metabolomics analyses were performed on serum samples to identify the differentially abundant metabolites between populations with and without MASLD. Liquid chromatography‒tandem mass spectrometry (LC‒MS/MS) analyses were performed using a UHPLC system (Vanquish, Thermo Fisher Scientific), and a QE HFX mass spectrometer was used for its ability to acquire MS/MS spectra in information-dependent acquisition (IDA) mode in the control of the acquisition software (Xcalibur, Thermo).

### Assessment of MASLD in humans

MASLD was defined based on the presence of hepatic steatosis, assessed by a fatty liver index (FLI) ≥ 60 in the UK Biobank or by abdominal ultrasonography in the SPECT-China. This diagnosis was further confirmed by meeting one of three additional criteria: overweight/obesity, diabetes, or metabolic dysregulation. Overweight/obesity was characterized by a body mass index (BMI) ≥ 25 kg/m^2^ in the UK Biobank or ≥ 24 kg/m^2^ in SPECT-China. Diabetes was identified through self-reported or hospital diagnoses, the use of antihyperglycemic medications or insulin, or a glycosylated hemoglobin A1c (HbA1c) > 6.5% (48 mmol/mol), with fasting blood glucose levels ≥ 7.0 mmol/L specific to the SPECT-China population. Metabolic dysregulation was defined by the presence of at least two of the following risk factors: increased waist circumference, elevated blood pressure, high triglyceride levels, low HDL-C, prediabetes, or elevated high-sensitivity C-reactive protein levels, as detailed in Table S1 (Eslam et al. 2020).

### Animals and treatment

All animals were maintained in a temperature-controlled room (22 °C ± 2 °C) under standard experimental conditions with a reversed 12:12 h dark:light cycle and free access to food and water. No rats or mice died during the experiment. All procedures were conducted in accordance with the guidelines of the Animal Research Committee of Shanghai Ninth People’s Hospital and Institutional Animal Care and Use Committee and conformed to the Animal Research: Reporting of In Vivo Experiments (ARRIVE) guidelines.

#### Rats

Twelve male Wistar rats (8 weeks of age) were purchased from Shanghai Laboratory Animal Centre in China and randomized into the control group (*n* = 6) or the L-Phe group (*n* = 6) after one week of acclimatization. Rats in both groups were fed a standard chow diet (P1101F-25, Shanghai Puluteng Biotechnology Co., Ltd., China), those in the control group were fed distilled water, and those in the L-Phe group were fed distilled water supplemented with 2 g/L L-Phe (Sigma, Aldrich, USA) (Pascucci et al. 2013). The intervention lasted for 28 weeks.

#### Mice

Twenty-four male C57BL/6 J mice (8 weeks of age) were purchased from GemPharmatech in China and randomly assigned to one of the following four groups: the control (*n* = 6), L-Phe (*n* = 6), vector + L-Phe (*n* = 6), and BNIP3 + L-Phe (*n* = 6) groups after one week of acclimatization. We administered adeno-associated virus serotype-8 (AAV8) vectors to 9-week-old mice via tail vein injection to overexpress BNIP3 (Genomeditech, Shanghai, China) in the liver at a dose of 3 × 10^11^ genome copies per mouse. After 10 weeks of age, the mice in the control group were fed distilled water, and in the L-Phe, vector + L-Phe, and BNIP3 + L-Phe groups, the mice were fed distilled water containing 2 g/L L-Phe. Mice in all groups were fed a standard chow diet (P1101F-25, Shanghai Puluteng Biotechnology Co., Ltd., China). The intervention lasted for 12 weeks.

### Body composition analysis of the animals

The body weights of the rats and mice were measured weekly, and the body composition was tested one week before sacrifice. All rats and mice were anaesthetized with gas with isoflurane (RWD Life science, China) and scanned with an Insight VET DXA (Biotimes Technology, China). In the DXA analysis, fat mass, lean mass, and total mass were measured, the ratio of fat to total mass was calculated, and images were generated (Ning et al. 2017).

### Targeted metabolomics and proteomics for animals

Serum samples were collected from animals under deep anesthesia induced by tail vein intravenous injection of a pentobarbital-dexmedetomidine mixture. The serum was subsequently isolated and stored at −80 °C. UHPLC-MS/MS analysis was performed using an Agilent 1290 Infinity II series (Agilent Technologies) to quantify 25 amino acids, including L-Phe. Briefly, a 15 μL sample was added to a 185 μL aliquot of the extraction solvent (acetonitrile-methanol–water, 80:80:25, containing an isotopically labelled internal standard mixture). The samples were vortexed for 30 s and sonicated for 15 min in an ice-water bath, followed by incubation at −40 °C for 1 h and centrifugation at 12,000 rpm and 4 °C for 15 min and then UHPLC-MS/MS. Mobile phase A was 1% formic acid in water, and mobile phase B was 1% formic acid in acetonitrile. The column temperature was set at 35 °C. The autosampler temperature was set at 4 °C, and the injection volume was 1 μL. Agilent MassHunter Work Station Software (B.08.00, Agilent Technologies) was used for multiple reaction monitoring data acquisition and processing.

TMT-based proteomic analysis was performed by Luming Biotech (Shanghai, China). In brief, the liver proteins of the rats were extracted from the lysates, the protease inhibitor PMSF was added, and the final concentration was 1 mM. The samples were ultrasonically crushed on ice and centrifuged at 12,000 rpm for 10 min at 4 °C. A total of 50 μg of protein from each sample was digested with trypsin for TMT labelling and proteomic analysis. The TMT-labelled peptides were fractionated by 1100 HPLC (Agilent) using an Agilent Zorbax Extend – C18 column (2.1 × 150 mm, 5 μm). LC‒MS/MS analysis was performed by a Triple TOF 5600 mass spectrometer equipped with a Nanospray III source (SCIEX, USA). The differentially expressed proteins were screened if their expression changed by > 1.2-fold (< 0.83-fold downregulation or > 1.2-fold upregulation), and *p* < 0.05 was considered to indicate statistical significance. All the gene models were subjected to the draft genome database for Kyoto Encyclopedia of Genes and Genomes (KEGG) and Gene Ontology (GO) annotation and classification.

### Biochemical evaluation and histological analyses of the animals

Intraperitoneal glucose tolerance tests (IPGTTs) and intraperitoneal insulin tolerance tests (IPITTs) were conducted one week before sacrifice. The detailed methods are consistent with our previous work (Wan et al. 2021). After sacrifice, biochemical indices, including TG, TC, HDL-C, LDL-C, ALT, and AST, were analysed in the serum samples of rats and mice by a Beckman Coulter AU 680 analyser (Beckman Coulter, Brea, CA).

Lipid accumulation was determined using Oil Red O staining and haematoxylin and eosin (H&E) staining. Liver tissues were formalin-fixed and paraffin-embedded, and 5 µm tissue sections were then prepared for staining with Sirius Red and H&E. Frozen liver sections were prepared in Tissue-Tek OCT compound to visualize lipid droplet accumulation using Oil Red O staining.

### Cell culture and treatment

The human hepatoblastoma cell line HepG2 was purchased from the Cell Bank of the Chinese Academy of Medical Sciences (catalogue number SCSP-510) and cultured in DMEM supplemented with 10% FBS, 100 U/ml penicillin and 100 mg/mL streptomycin. All cell culture reagents were purchased from Gibco (Grand Island, NY, USA). L-Phe was added to the medium to achieve final concentrations of 1, 2, 4, 6 and 8 mM (Zhou et al. 2022). Based on the screening results, 8 mM L-Phe treatment for 24 h was used for mechanistic research. For lentivirus infection, cells were incubated with a lentivirus overexpressing BNIP3 (NM_004052.4) and selected with puromycin to construct stable cell lines overexpressing BNIP3 following the manufacturer’s protocol. The lentiviruses were purchased from Genomeditech Corporation (Shanghai, China).

Cultured cells were grouped by treatment with rapamycin (RAPA, 50 nM), L-Phe, or RAPA + L-Phe (50 nM RAPA for 12 h and then cultured with 8 mM L-Phe). Negative control cells and BNIP3-overexpressing cells were grouped by treatment with or without L-Phe. The cells were incubated with medium containing L-Phe for 24 h before harvesting.

### Assessment of apoptosis by TUNEL assay

Paraffin-embedded liver sections were deparaffinized, rehydrated, and subjected to proteinase K digestion (20 μg/mL, 15 min). Apoptotic cells were detected using a TUNEL assay kit (Servicebio, China), following the manufacturer’s protocol. Nuclei were counterstained with DAPI. TUNEL-positive cells (green fluorescence) were quantified in ≥ 5 random fields per sample using ImageJ.

### Assessment of cell viability by Cell Counting Kit-8 (CCK8) assay

Cell viability was assessed using the CCK-8 assay (CK04, Dojindo Laboratories, Japan). HepG2 cells were seeded into 96-well plates at a density of 2000 cells per well. Upon reaching 50% confluence, cells were treated with varying concentrations of L-Phe for 24 h, followed by three PBS washes. For the CCK-8 assay, 10 μL of CCK-8 solution was added to each well, and the plates were incubated at 37 °C for 1 h. The absorbance was measured at 450 nm using a microplate reader. Cell viability was calculated as a percentage of the control group (untreated cells). Each experiment was performed in triplicate. One-way ANOVA followed by Tukey’s post-hoc test to determine significant differences.

### Assessment of mitophagy levels in cells

Detection of the mitochondrial membrane potential (MMP) was performed via JC-1 staining (Beyotime, China). According to the manufacturer’s instructions, the cells were incubated with JC-1 dye for 20 min at 37 °C and then washed with JC-1 staining buffer. The JC-1 aggregates that formed red fluorescence showed a high MMP, while the JC-1 monomers that formed green fluorescence showed a low MMP. The fluorescence intensities of mitochondrial JC-1 monomers and aggregates were observed and imaged under a fluorescence microscope (Nikon, Japan) and quantified by ImageJ.

Cultured cells were incubated with 50 nM MitoTracker Green and 50 nM LysoTracker Red (Beyotime, China) for 30 min at 37 °C to monitor the levels of mitochondria and lysosomes, respectively. After incubation and replacement of the medium with fresh medium, the cells were immediately imaged by confocal microscopy (Nikon, Japan) and quantified by ImageJ.

### Immunohistochemistry (IHC) and Immunofluorescence (IF)

Paraffin-embedded liver sections were subjected to immunoassays. After the sections were incubated in 3% H_2_O_2_ for 30 min at 37 °C to quench endogenous peroxidase activity, they were subjected to antigen retrieval in a microwave and then blocked with 5% BSA for 1 h at room temperature. The sections were labelled with the primary antibody for 3 h, incubated with horseradish peroxidase-conjugated secondary antibodies for 1 h, mounted with DAPI-containing mounting medium and then viewed under a fluorescence microscope.

For immunofluorescence, paraffin-embedded liver sections were subjected to antigen retrieval in a microwave and then blocked with 5% BSA for 30 min at room temperature. Cultured cells were collected and then fixed with 4% formaldehyde for 30 min, permeabilized with 0.1% Triton X-100 in PBS for 5 min, and blocked with 10% goat serum in PBS for 30 min. Paraffin-embedded sections and cultured cells were incubated with primary antibodies and secondary antibodies. Nuclei were counterstained with DAPI. Images were captured using a Nikon microscope and quantified by ImageJ.

### Quantitative real-time PCR

Total RNA was extracted from liver tissues or HepG2 cells using TRIzol reagent (TAKARA, Japan). Total RNA was reverse-transcribed to cDNA in a 20 mL reaction using a reverse transcription kit (TAKARA, Japan). SYBR Green Master Mix (Yeasen, China) was used for qPCR. The whole process was carried out according to the guidelines of the kits. The relative abundance of mRNA of target genes was quantified by the 2^−△△CT^ method and normalized to that of β-actin. The primers used are shown in Table S2.

### Western blot

Liver tissues or HepG2 cells were homogenized and lysed with RIPA buffer (Solarbio, China) containing PMSF protease inhibitor (Solarbio, China) at 4 °C and then denatured by boiling for 10 min with SDS. The protein samples were subjected to SDS‒PAGE and transferred to PVDF membranes. The membranes were blocked with 5% skim milk in TBST for 1 h at room temperature and incubated with primary antibodies (Table S3) overnight at 4 °C. The membranes were washed three times with TBST and then incubated with the corresponding secondary antibody diluted in blocking buffer at 37 °C for 1 h. The bands were detected using a chemiluminescent imaging system and quantified by ImageJ software.

### Transmission electron microscopy (TEM)

Liver tissues or cultured HepG2 cells were fixed in 2.5% glutaraldehyde overnight at 4 °C. Then, the cells were postfixed in 1% osmium tetroxide for 1 h, dehydrated with ethanol, and polymerized using epoxy resin. Ultrathin sections were cut and stained with aqueous uranyl acetate and lead citrate. Finally, mitochondrial morphology and mitophagy were observed via a JEM-1400 transmission electron microscope (JEOL, Tokyo, Japan).

### Statistical analyses

All the statistical analyses were conducted with IBM SPSS Statistics version 26 (IBM Corporation, USA) and GraphPad Prism version 9.0 (GraphPad, Software). Student’s two-sided t test or analysis of variance (ANOVA) with Tukey post hoc test was used to test the differences between two or multiple groups, and chi-square tests were used for categorical variables. Linear regression and logistic regression analyses were used to estimate the β or odds ratio (OR) and 95% confidence interval (CI). Potential confounders adjusted in the models are listed in the legends.

## Results

### Increased plasma/serum L-Phe is related to deteriorated lipid metabolism indicators and higher prevalence of MASLD in European and Chinese populations

**Fig. 1:**
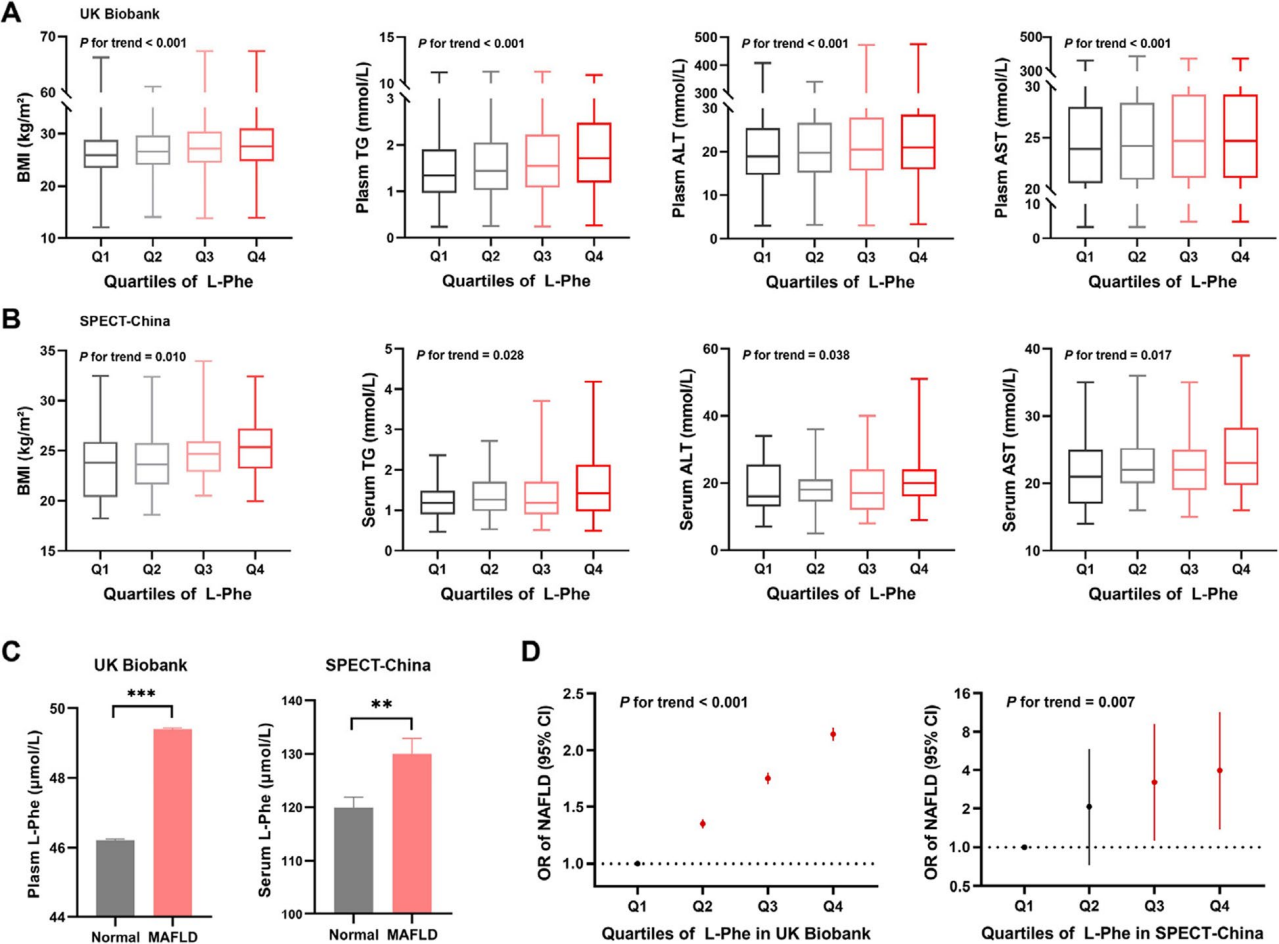
Increased plasma/serum L-Phe is related to lipid metabolism indicators and the prevalence of MASLD in the UK Biobank and SPECT-China populations. **A** Associations between serum triglycerides (TG), aspartate aminotransferase (ALT), alanine aminotransferase (AST) and quartiles of plasma L-Phe in the UK Biobank population. A linear regression model was used, with continuous variables grouped by quartiles are used as independent variables. **B** Associations between serum triglycerides (TG), aspartate aminotransferase (ALT), and alanine aminotransferase (AST) by quartiles of serum L-Phe in the SPECT-China population. A linear regression model was used, with continuous variables grouped by quartiles are used as independent variables. Q, quartile. **C** L-Phe levels in plasma for individuals with and without MASLD in the UK Biobank population (left, *n* = 183,482) and L-Phe levels in serum for individuals with and without MASLD in the SPECT-China population (right, *n* = 155). Two-tailed Student’s t test was used for two-group comparisons. The data are presented as the means ± SEMs, ** *p* < 0.01, *** *p* < 0.001. **D** Adjusted odds ratios (95% confidence intervals) for MASLD across quartiles of plasma L-Phe in the UK Biobank (left) and serum L-Phe in the SPECT-China population (right). Logistic regression models were adjusted for age, sex, race, Townsend deprivation index, education, current smoking, and ideal physical activity in the UK Biobank study and adjusted for age, sex, urban, education, current smoking, and ideal physical activity in the SPECT-China study

The general characteristics of the participants in the UK Biobank and SPECT-China according to the quartiles of L-Phe are shown in Tables S4 and S5. Compared with individuals in the lowest quartile of L-Phe, UK Biobank participants in the higher quartiles of L-Phe had increased levels of weight, ALT, AST, FLI, waist circumference (WC), BMI, γ-glutamyl transferase alanine and TG (Fig. 1A and Table S4, *p* for trend < 0.001). Similarly, in the SPECT-China population, increased weight, BMI, TG, ALT, and AST were also associated with increasing quartiles of L-Phe (Fig. 1B and Table S5, *p* for trend < 0.05). The prevalence of MASLD was 38.1% in the UK Biobank and 41.9% in SPECT-China. Compared to non-MASLD individuals, patients with MASLD had significantly greater L-Phe levels in both populations (Fig. 1C, *p* < 0.05). After adjusting for confounders, a significant association was observed between the highest quartile of L-Phe and MASLD (OR, 95% CI: 2.14, 2.08–2.20 for the UK Biobank and 3.98, 1.38–11.45 for SPECT-China; both *p* values for trend < 0.05) and between each 1 SD increase in L-Phe and MASLD (Fig. 1D and Table S6, both* p* < 0.05). These results demonstrate that elevated levels of L-Phe are independently associated with increased odds of MASLD in humans.

### L-Phe regulates lipid metabolism and exacerbates liver steatosis

**Fig. 2:**
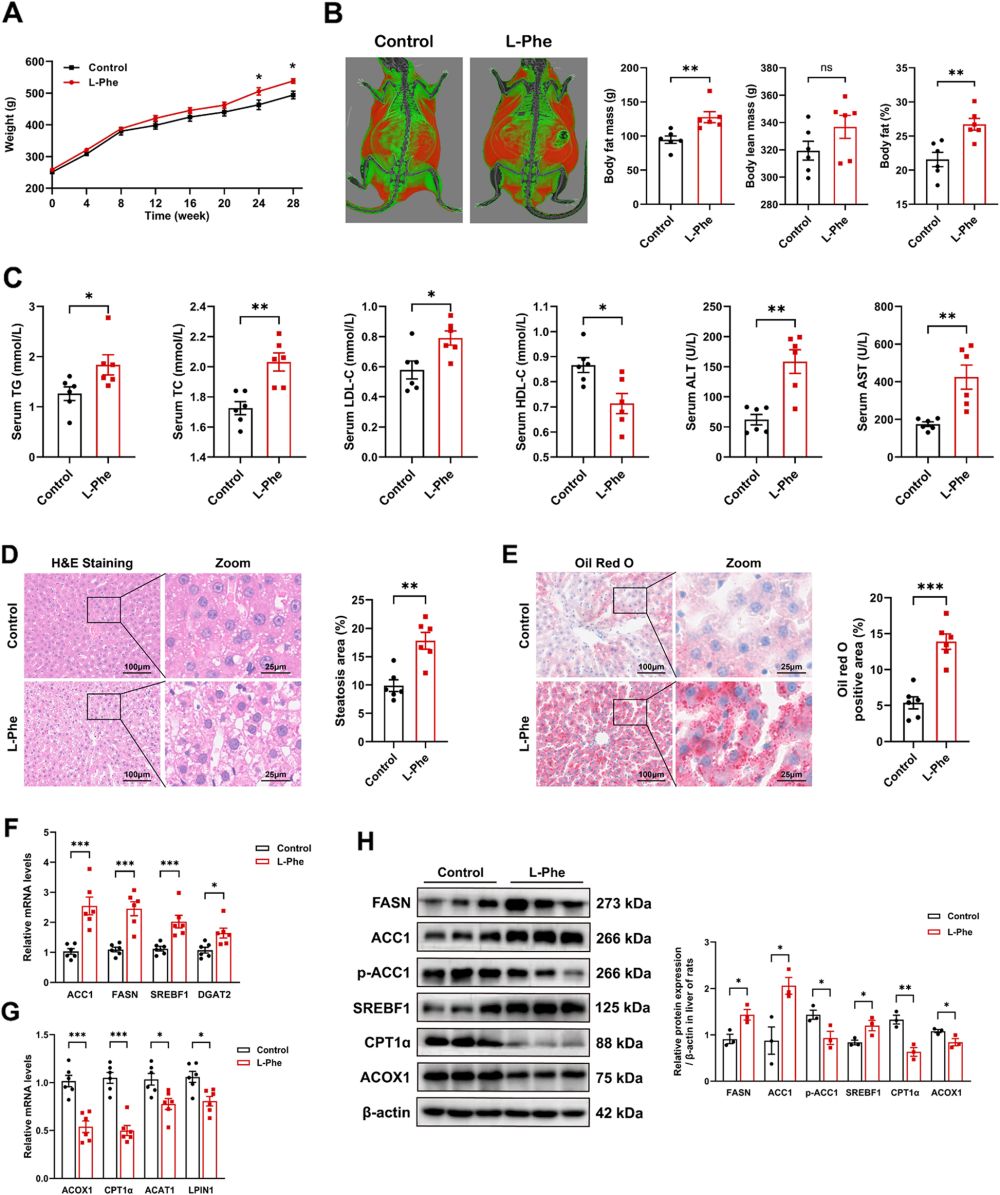
L-Phe regulates lipid metabolism and exacerbates liver steatosis. **A** Body weight of rats over the 28-week intervention period (*n* = 6 per group). **B** Representative body composition image (left) and quantification of fat mass, lean mass, and fat percentage of rats in week 27 (*n* = 6 per group). **C** Serum triglyceride (TG), total cholesterol (TC), low-density lipoprotein cholesterol (LDL-C), high-density lipoprotein cholesterol (HDL-C), aspartate aminotransferase (ALT), and alanine aminotransferase (AST) levels in the rats (*n* = 6 per group). **D** H&E staining (left) and steatosis quantification (right) of liver sections from the rats (*n* = 6 per group). **E** Oil Red O staining (left) and quantification (right) of liver sections from the rats (*n* = 6 per group). **F** Relative mRNA levels of lipogenesis-related genes in the livers of the rats (*n* = 6 per group). **G** Relative mRNA levels of fatty acid decomposition-related genes in the livers of rats (*n* = 6 per group). **H** Western blot analysis (left) and quantification (right) of proteins involved in fatty acid metabolism in the livers of rats (*n* = 3 per group). The data are presented as the means ± SEMs. Two-tailed Student’s t test was used for two-group comparisons. *p < 0.05, **p < 0.01, ***p < 0.001, ns, not significant

We speculated that high levels of L-Phe could lead to lipid metabolism disorders and contribute to the onset of MASLD. Thus, we established a rat model of L-Phe exposure by administering water containing 2 g/L L-Phe according to a previous study (Pascucci et al. 2013). Western blot analysis reveals that LAT1, a receptor for L-Phe, was upregulated in both liver of rats and HepG2 cell lines following L-Phe treatment compared to the Control group (Figure S2A). The concentrations of L-Phe in serum and liver were elevated in the L-Phe group compared to those in the Control group, indicating successful modelling (Figure S2B, *p* < 0.05). During weeks 24 to 28 of exposure, the L-Phe group exhibited greater body weight than did the Control group (Fig. 2A, *p* < 0.05). Body composition analysis revealed a significant increase in fat mass and percentage of fat in the L-Phe group compared to the Control group (Fig. 2B, *p* < 0.001), while no significant difference in lean mass was detected (*p* > 0.05). In addition, biochemical indicators associated with MASLD were evaluated, and rats in the L-Phe group had significantly increased TG, TC, LDL-C, AST, and ALT levels and decreased HDL-C levels compared to those in the Control group (Fig. 2C, *p* < 0.05). However, the results of the fasting blood glucose test, intraperitoneal glucose tolerance tests and intraperitoneal insulin tolerance tests showed no significant differences between the L-Phe and Control groups (Figure S2C-S2E, *p* > 0.05).

H&E staining of liver sections from the Control group revealed clear hepatocyte cytoplasm with fewer lipid vacuoles. Conversely, abundant lipid vacuoles were observed within the hepatocyte cytoplasm in the L-Phe group (Fig. 2D, *p* < 0.01). Subsequent Oil Red O staining confirmed these findings, revealing fewer lipid deposits in the control liver than in the L-Phe group, and markedly increased and deeper-colored positive vacuoles were detected in the L-Phe group (Fig. 2E, *p* < 0.001). These results suggest that L-Phe treatment potentially induces hepatic lipid accumulation, supporting its role in hepatic steatosis. Immunofluorescence analysis revealed a significant increase in CD68 macrophage infiltration in the livers of L-Phe group compared to Control group (Figure S2F, *p* < 0.05). Additionally, L-Phe exposure triggered an upregulation of key pro-inflammatory mediators including TNFα and IL-1β, while the increment in MCP1 and iNOS did not achieve the statistically significant (Figure S2G). And the size of visceral adipocytes in visceral adipose tissue was greater (Figure S2I, *p* < 0.05). The expression of genes related to lipogenesis, including acetyl‐CoA carboxylase 1 (ACC1), fatty acid synthase (FASN), sterol regulatory element-binding transcription factor 1 (SREBF1), and diacylglycerol O-acyltransferase 2 (DGAT2), was upregulated, and the expression of genes related to fatty acid β-oxidation, including acyl-CoA oxidase 1 (ACOX1), carnitine palmitoyl transferase 1α (CPT1α), acetyl-CoA acetyltransferase 1 (ACAT1), and LPIN1, was reduced in the livers of the L-Phe group compared to those of the Control group (Fig. 2F and G, *p* < 0.05). Similarly, western blot analysis reveals that hepatic de novo lipogenesis‐related proteins ACC1 and FASN and the related transcription factor SREBF1 were significantly increased by L-Phe feeding, and p-ACC1 (Ser79), ACOX1, and CPT1α were significantly reduced (Fig. 2H, *p* < 0.05). Together, these findings indicate that elevated L-Phe may promote liver steatosis through the impairment of fatty acid metabolism.

### L-Phe suppresses mitophagy in liver steatosis

**Fig. 3:**
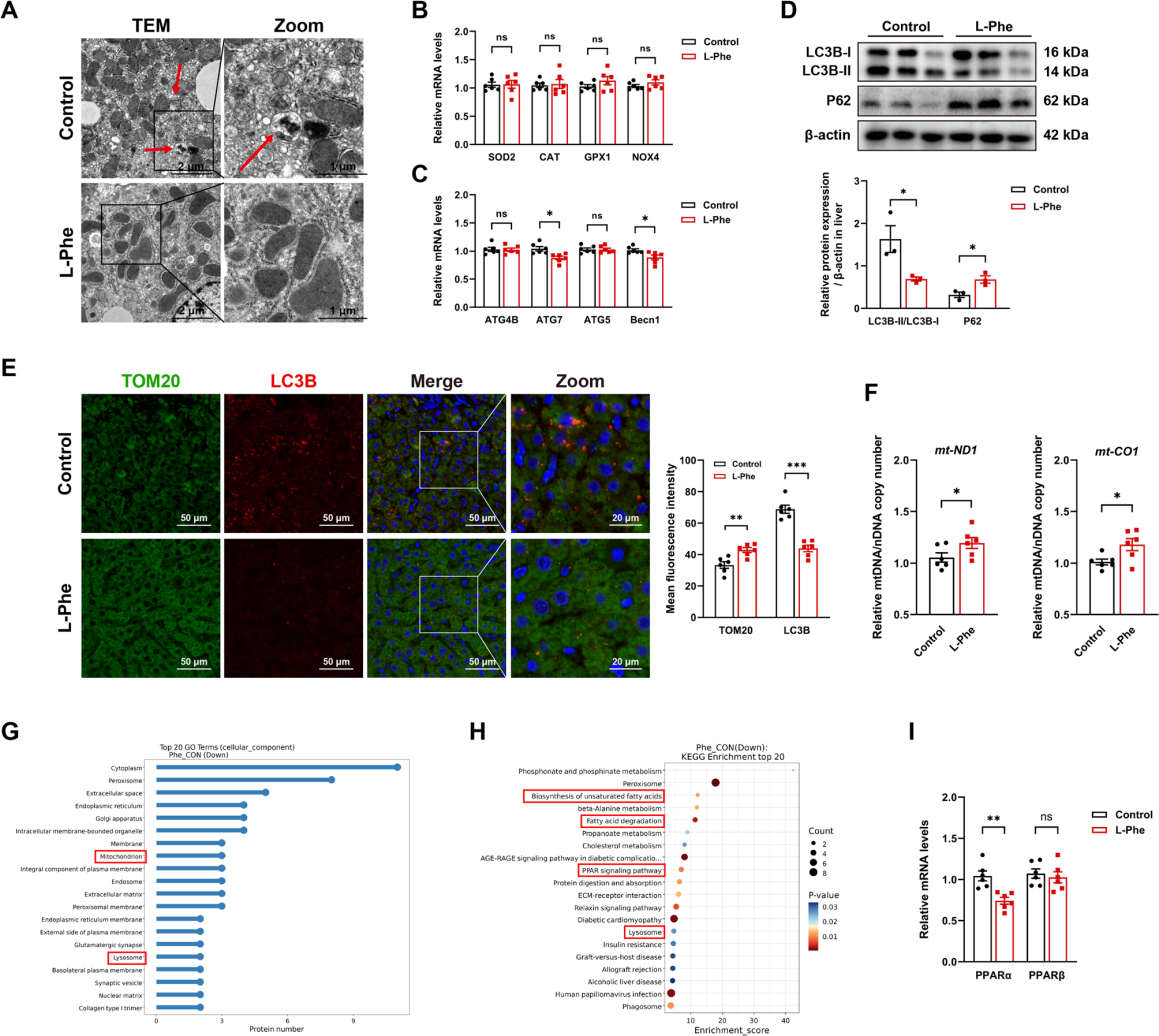
L-Phe suppresses mitophagy in liver steatosis.A Transmission electronic microscopy analysis of mitochondrial structure in the livers of rats (n=6 per group). The red arrow represents mitophagosomes. B Relative mRNA expression of genes related to mitochondria ROS (n=6 per group). C Relative mRNA expression of genes related to mitophagy (n=6 per group). D Western blot analysis (up) and quantification (down) of mitophagy-related proteins in the livers of the rats (n=3 per group). E Immunofluorescence staining (left) and quantification (right) of TOM20 (green), LC3B (red) in liver sections from rats (n=6 per group). The cell nuclei were stained with DAPI (blue). F Relative mitochondrial DNA (mtDNA) copy number normalized to nuclear DNA (nDNA) in rat liver tissues. mtDNA levels were quantified by qPCR using primers for mitochondrial genes (mt-ND1 and mt-CO1) and normalized to the nuclear β-actin gene (n=6 per group). G Gene ontology (GO) analysis of cellular components in the livers of rats (n=4 per group). H Kyoto Encyclopaedia of Genes and Genomes (KEGG) pathway enrichment of the differentially expressed proteins in the livers of the rats (n=4 per group). I Relative mRNA expression of PPARα and PPARβ in liver of rats were determined using qPCR (n=5-6 per group). The data are presented as the means ± SEMs. Two-tailed Student’s t test was used for two-group comparisons. *p < 0.05, **p < 0.01, ***p < 0.001, ns, not significant.

To gain further insight into the role of L-Phe in liver steatosis, TEM was performed on the liver to observe possible structural or functional alterations at the organelle level. The Control group has distinct internal structures, while the L-Phe group has a reduced electron density and appears to be blurred (Fig. 3A). More importantly, in the surrounding irregular or swollen mitochondria, there was a notable absence of typical autophagic structures such as autophagosomes or autolysosomes. The crucial role of mitophagy, wherein mitochondria fuse with lysosomes to form autolysosomes for subsequent degradation, has been established (Undamatla et al. 2023), indicating that reduced mitophagy is an early feature of MASLD (Palikaras et al. 2018). To evaluated the function of mitochondria, we analysed the key genes related to ROS and mitophagy (Fig. 3B and C). Compared to the Control group, the expression of ATG7 and Becn1 were decreased in L-Phe group (Fig. 3C, *p* < 0.05). The protein expression levels of LC3BII/I were downregulated, and the expression of P62 was upregulated in the livers of the L-Phe group (Fig. 3D, *p* < 0.05). Immunofluorescence staining for LC3B and TOM20 further confirmed the significantly increased TOM20 staining and reduced overlap of LC3B-TOM20 staining in the L-Phe group compared to the Control group (Fig. 3E, *p* < 0.001). Moreover, L-Phe group exhibited a significant increment in relative mtDNA/nDNA compared to the Control group (Fig. 3F, *p* < 0.05). These findings indicated that L-Phe could inhibit mitophagy, potentially result in mitochondrial accumulation and contribute to the dysfunction of lipid metabolism. However, L-Phe treatment exerted no significant effects on hepatocyte apoptosis rates evaluated by TUNEL assay or the expression levels of cleaved caspase-3 (Figure S3A-S3C, *p* > 0.05). At the same time, GO analysis of cellular components revealed the downregulation of mitochondria and lysosomes in the L-Phe group (Fig. 3G). KEGG pathway enrichment analysis of the differentially expressed proteins in the livers of the two groups revealed that exposure to L-Phe significantly reduced pathways related to PPARα and unsaturated fatty acid biosynthesis (Fig. 3H and I), where deficiency of unsaturated fatty acids has been linked to impaired mitophagy and the progression of liver steatosis in MASLD (Spooner and Jump 2023; Gortan Cappellari et al. 2022; Li et al. 2024). These results support that L-Phe downregulates mitophagy, which may thereby promote liver steatosis.

### L-Phe promotes liver steatosis through inhibiting mitophagy

We preestablished an in vitro model of HepG2 cells treated with different concentrations of L-Phe. Results from the CCK-8 assays showed that within the tested concentration range, L-Phe did not significantly impact cell viability (Figure S4A, *p* > 0.05). L-Phe supplementation for 24 h upregulated the protein expression of ACC1, FASN, and SREBF1 and downregulated the protein expression of p-ACC1 (Ser79), CPT1α and ACOX1 in a dose-dependent manner, with 8 mM L-Phe treatment having the strongest effects on increasing fatty acid synthesis and decreasing β-oxidation (Figure S4B, *p* < 0.05). Western blot analysis revealed that treatment with a high concentration of L-Phe resulted in greater downregulation of BNIP3 and LC3B-II/I protein expression and greater upregulation of P62 (Figure S4C, *p* < 0.05). An increase in the mitochondrial membrane potential (MMP) was observed when the concentration of L-Phe was greater than 2 mM, and the green fluorescence of the JC-1 monomer was almost invisible in the L-Phe 8 mM treatment group (Figure S4D, *p* < 0.05). Moreover, the level of mitochondrial and lysosomal colocalization decreased with increasing levels of L-Phe (Figure S4E, *p* < 0.05), suggesting that 8 mM L-Phe was suitable for further experiments.

**Fig. 4:**
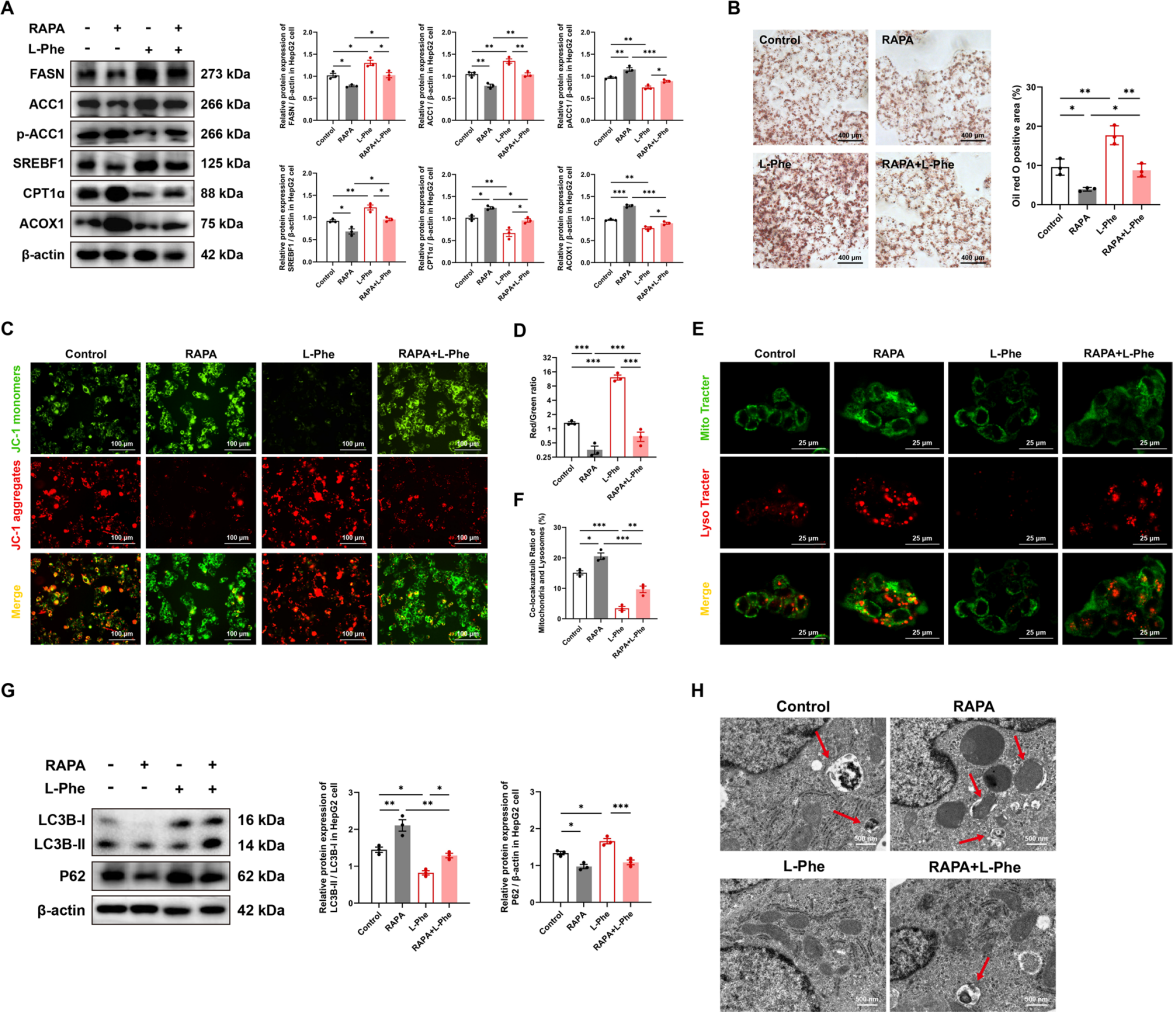
L-Phe promotes liver steatosis through mitophagy. **A** Western blot analysis (left) and quantification (right) of proteins involved in fatty acid metabolism in HepG2 cells treated with rapamycin and/or L-Phe (*n* = 3 per group). **B** Oil Red O staining (left) and quantification (right) of HepG2 cells treated with rapamycin and/or L-Phe (*n* = 3 per group). **C** and **D** JC-1 staining (**C**) and quantification (**D**) of the mitochondrial membrane potential in HepG2 cells. The ratio is the JC-1 aggregate (red) to monomer (green) ratio (*n* = 3 per group). **E** and **F** Colocalization of mitochondria (green) and lysosomes (red) labelled with MitoTracker Green and LysoTracker Red (**E**), respectively, and the percentage of yellow puncta colabelled with MitoTracker Green and LysoTracker Red (**F**) (*n* = 3 per group). **G** Western blot analysis and quantification of proteins involved in mitophagy in HepG2 cells treated with rapamycin and/or L-Phe (*n* = 3 per group). **H** Transmission electron microscopy analysis of mitochondrial structure (left) and quantification (right) for HepG2 cells treated with rapamycin and/or L-Phe (*n* = 5 per group). The red arrow represents mitophagosomes. The data are presented as the means ± SEMs. Two-tailed Student’s t test was used for two-group comparisons. *p < 0.05, **p < 0.01, ***p < 0.001, ns, not significant

To clarify whether the mechanism by which L-Phe promotes liver steatosis is associated with mitophagy, we applied the mitophagy inducer RAPA to HepG2 cells and then treated them with or without L-Phe. Compared with L-Phe treatment, RAPA pretreatment decreased the expression of ACC1, FASN, and SREBF1 and increased the expression of p-ACC1 (Ser79), CPT1α and ACOX1 in cells (Fig. 4A, *p* < 0.05). Consistent with this, HepG2 cells treated with RAPA and L-Phe showed reduced steatosis compared to those treated with L-Phe alone, as indicated by the reduction in red lipid droplets (Fig. 4B, *p* < 0.05). In addition, RAPA treatment activated L-Phe-induced mitophagy inhibition, with a decrease in the MMP (Fig. 4C and D, *p* < 0.001), increased colocalization of mitochondria with lysosomes (Fig. 4E and F, *p* < 0.01), upregulated expression of LC3II/I, and downregulated expression of P62 (Fig. 4G, *p* < 0.05). TEM analysis revealed that the number of mitophagosomes increased in HepG2 cells treated with RAPA but decreased in those treated with L-Phe (Fig. 4H, *p* < 0.01). However, it should be acknowledged that RAPA, also as an mTOR inhibitor, can exert an effect on the process of mitophagy by inhibiting the mTOR pathway to inhibit lipid accumulation in liver. Therefore, although the above data suggest that L-Phe may promote lipid accumulation by inhibiting mitophagy, further studies are needed to verify its role by intervening with targeted molecules.

### BNIP3 overexpression activates mitophagy in L-Phe-induced hepatic steatosis

**Fig. 5:**
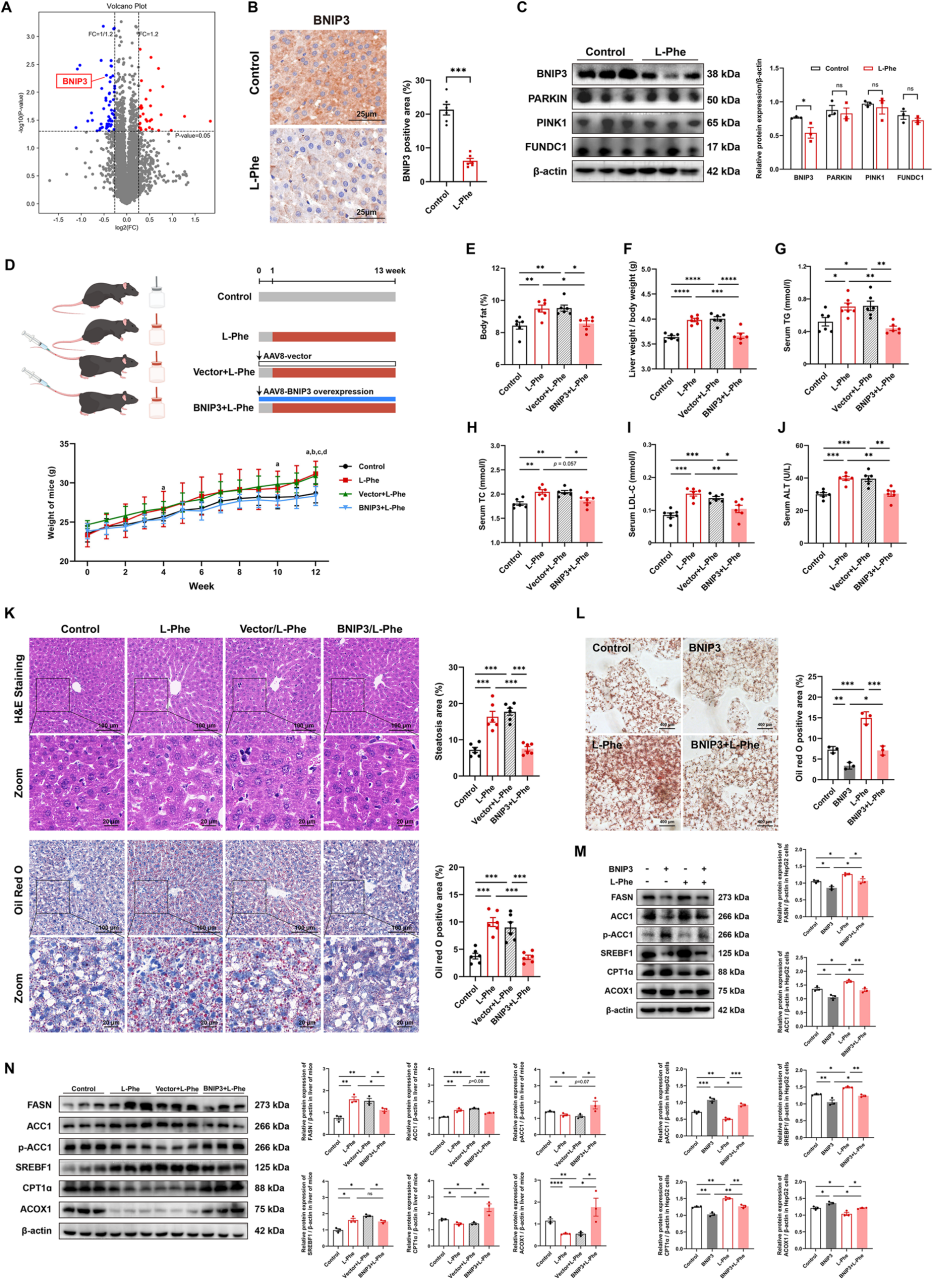
L-Phe induced hepatic steatosis by regulating BNIP3. **A** Volcano plot of the differentially expressed proteins in the livers of the rats (*n* = 4 per group). **B** Immunohistochemical staining for BNIP3 (left) and quantification (right) of liver sections from the rats (*n* = 6 per group). **C** Western blot analysis (left) and quantification (right) of mitophagy-related proteins in rats (*n* = 3 per group). **D** Schematic illustration showing the experimental design for BNIP3 overexpression via tail vein injection of AAV-8 and L**-**Phe (top). Body weights of the mice over the 12-week intervention period (bottom) (*n* = 6 per group). a, Vector + L-Phe compared to BNIP3 + L-Phe, *p* < 0.05; b, Control compared to L-Phe, *p* < 0.05; c, Control compared to Vector + L-Phe, *p* < 0.05; d, L-Phe compared to BNIP3 + L-Phe, *p* < 0.05. **E** Liver fat percentage in the mice (*n* = 6 per group). **F** Liver weight/body weight ratio of the mice (*n* = 6 per group). **G**-**J** Serum triglyceride (TG), total cholesterol (TC), low-density lipoprotein cholesterol (LDL-C), and aspartate aminotransferase (ALT) levels in the mice (*n* = 6 per group). **K** H&E staining and steatosis quantification (top) and Oil Red O staining and quantification (bottom) of liver sections from mice (*n* = 6 per group). **L** Oil Red O staining (left) and quantification (right) of HepG2 cells with BNIP3 overexpression and/or L-Phe treatment (*n* = 3 per group). **M** Western blot analysis and quantification of mitophagy-related proteins in HepG2 cells with BNIP3 overexpression and/or L-Phe treatment (*n* = 3 per group). **N** Western blot analysis (left) and quantification (right) of proteins involved in mitophagy in the livers of mice (*n* = 3 per group). The data are presented as the means ± SEMs. Two-tailed Student’s t test was used for two-group comparisons. One-way ANOVA with Tukey post hoc test was used for multigroup comparisons. *p < 0.05, **p < 0.01, ***p < 0.001, ns, not significant

Cluster analysis of differentially expressed proteins in the liver between the two groups revealed significant downregulation of the mitophagy-related protein BNIP3 in the L-Phe group compared to the control group (Fig. 5A). IHC staining confirmed that L-Phe downregulated the expression of BNIP3 in the liver (Fig. 5B, *p* < 0.001). Western blot analysis consistently demonstrated decreased BNIP3 expression in the L-Phe group (Fig. 5C, *p* < 0.001), but no significant changes in PINK1, PARKIN, or FUNDC1 expression were detected (*p* > 0.05).

We speculated that BNIP3-mediated mitophagy mediates the pathological process of liver steatosis. Thus, we used AAV8 to overexpress BNIP3 in mice, particularly in the liver, and empty AAV8 (vector) was injected as a reference, followed by treatment with L-Phe for 12 weeks. The transduction efficiency of BNIP3 was confirmed by western blotting and IHC after 3 months (Figure S5A and S5B,* p* < 0.01), and the serum L-Phe concentration was tested by targeted metabolomics (Figure S5C and S5D,* p* < 0.05). There was no significant difference between the four groups in the first 10 weeks for weight or food intake after AAV8 infection or L-Phe feeding (Fig. 5D and Figure S5E,* p* > 0.05). However, at the 12th week, L-Phe-induced weight gain was markedly ameliorated in the BNIP3 + L-Phe group (Fig. 5D, *p* < 0.01). The body fat percentage, liver-to-body weight ratio, and fat mass in the BNIP3 + L-Phe group were lower than those in the L-Phe and vector groups (Fig. 5E and F and Figure S5E,* p* < 0.05), while lean mass did not significantly differ among the four groups (Figure S5F,* p* > 0.05). In addition, BNIP3 overexpression decreased the TG, LDL-C, and ALT levels in the BNIP3 + L-Phe group compared with those in the vector group (Fig. 5G-J, *p* < 0.05). However, no significant differences in HDL-C, AST, FPG, or IPGTTs were detected among the groups (Figure S5G-S5J,* p* > 0.05). Hepatic lipid accumulation was markedly lower in the BNIP3 + L-Phe group than in the group without BNIP3 overexpression, as evidenced by H&E staining and Oil Red O staining of the liver (Fig. 5K, *p* < 0.001). Moreover, we established a BNIP3 overexpression model using lentivirus in vitro in HepG2 cells. Consistent with the in vivo results, there was less steatosis in the BNIP3 + L-Phe group than in the L-Phe group (Fig. 5L, *p* < 0.05). Western blot analysis revealed that L-Phe-induced increases in the expression of enzymes involved in fatty acid synthesis (ACC1, FASN, and SREBF1) were markedly attenuated, and low levels of p-ACC1 (Ser79) and genes involved in fatty acid β-oxidation (ACOX1 and CPT1α) were upregulated both in vitro and in vivo (Fig. 5M and N, *p* < 0.05). These results indicated that L-Phe-induced hepatic steatosis is mediated by the level of BNIP3.

### BNIP3 overexpression activates L-Phe-mediated inhibition of mitophagy

**Fig. 6:**
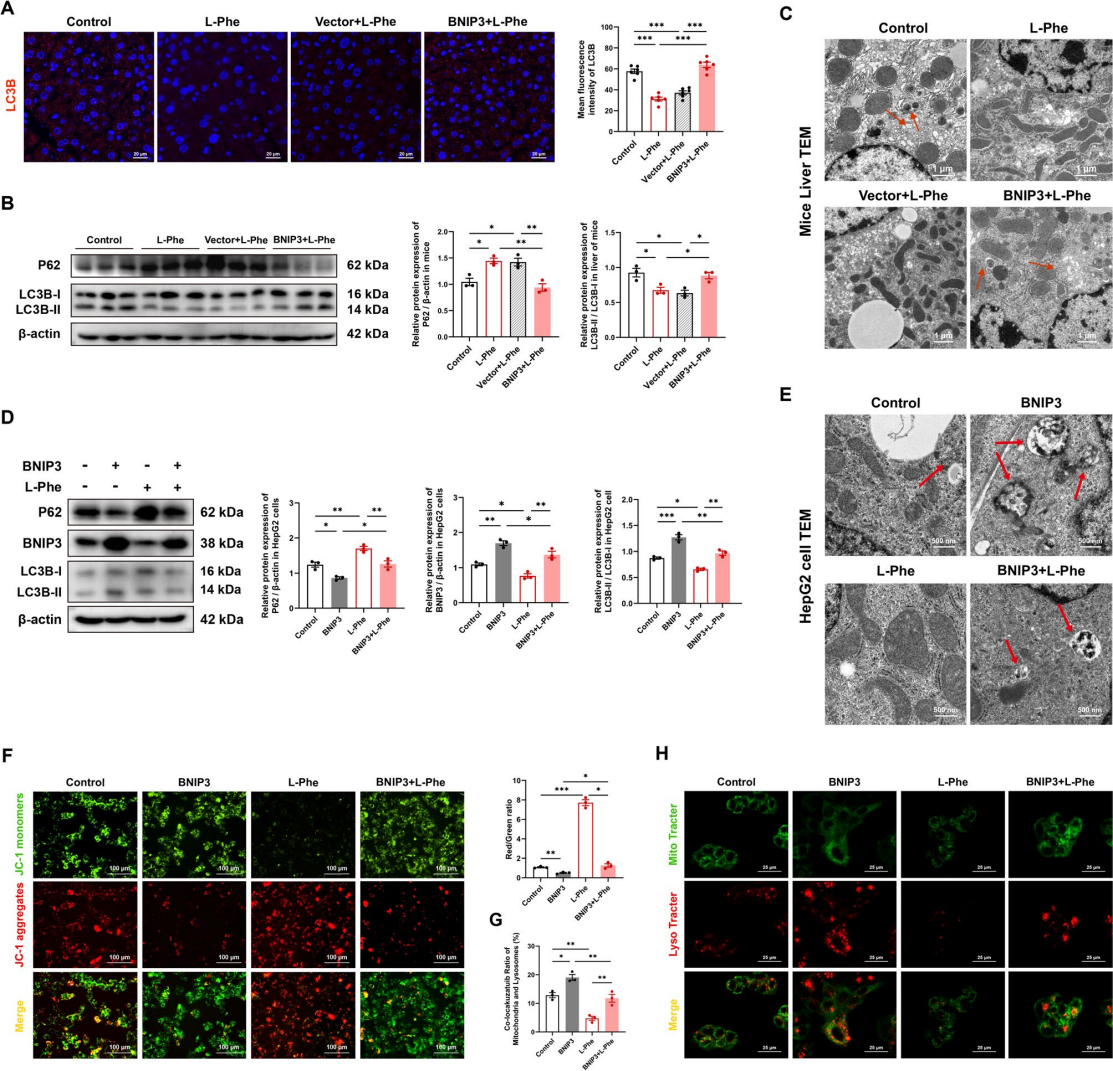
L-Phe induced hepatic steatosis through BNIP3-mediated mitophagy. **A** Immunofluorescence staining (left) and quantification (right) of LC3B (red) and DAPI (blue) in liver sections from mice (*n* = 6 per group). **B** Western blot analysis (left) and quantification (right) of the protein expression of P62 and LC3B in the livers of the mice (*n* = 3 per group). **C** Transmission electronic microscopy analysis of mitochondrial structure in the livers of mice (*n* = 4 per group). The red arrow represents mitophagosomes. **D** Western blot analysis and quantification of mitophagy-related proteins in HepG2 cells with BNIP3 overexpression and/or L-Phe treatment (*n* = 3 per group). **E** Transmission electron microscopy analysis of the mitochondrial structure (left) and quantification (right) of HepG2 cells overexpressing BNIP3 and/or subjected to L-Phe treatment. The red arrow represents mitophagosomes (*n* = 5 per group). **F** JC-1 staining (left) and quantification (right) of the mitochondrial membrane potential in HepG2 cells overexpressing BNIP3 and/or subjected to L-Phe treatment. The ratio is the JC-1 aggregate (red) to monomer (green) ratio (*n* = 3 per group). **G** and **H** Colocalization of mitochondria (green) and lysosomes (red) labelled with MitoTracker Green and LysoTracker Red (**H**), respectively, and the percentage of yellow puncta colabelled with MitoTracker Green and LysoTracker Red (**G**) (*n* = 3 per group). The data are presented as the means ± SEMs. One-way ANOVA with Tukey post hoc test was used for multigroup comparisons. *p < 0.05, **p < 0.01, ***p < 0.001

We further investigated whether BNIP3-mediated mitophagy is involved in L-Phe-induced liver steatosis. In vivo, overexpression of BNIP3 in the livers of mice increased the fluorescence intensity of LC3B after treatment with L-Phe (Fig. 6A, *p* < 0.001). Compared with those in the control group, the expression of P62 in the liver was greater and the LC3BII/I ratio was lower after L-Phe feeding, which was consistent with the results in rats and HepG2 cells (Fig. 6B, D, S4C, and 4G,* p* < 0.05). After BNIP3 was overexpressed both in vivo and in vitro, the regulatory effects of L-Phe were not significant; BNIP3 downregulated the increase in P62 expression and upregulated the decrease in LC3BII/I (Fig. 6B and D, *p* < 0.05). Then, we investigated mitochondria at the organelle level by TEM (Fig. 6C and E). In the BNIP3 overexpression model, the percentage of mitophagy decreased in response to L-Phe, as indicated by matrix swelling and vacuoles (*p* < 0.05). JC-1 staining revealed that the MMP of HepG2 cells in the BNIP3 + L-Phe group was greater than that in the BNIP3 group and lower than that in the L-Phe group (Fig. 6F, *p* < 0.05). BNIP3 overexpression increased the colocalization of L-Phe in mitochondria and lysosomes (Fig. 6G, *p* < 0.01). These results indicated that L-Phe-induced hepatic steatosis develops through inhibition of BNIP3-mediated mitophagy.

### L-Phe regulates the BNIP3-mediated PPARα and AMPK/mTOR signalling pathways to promote hepatic steatosis

**Fig. 7:**
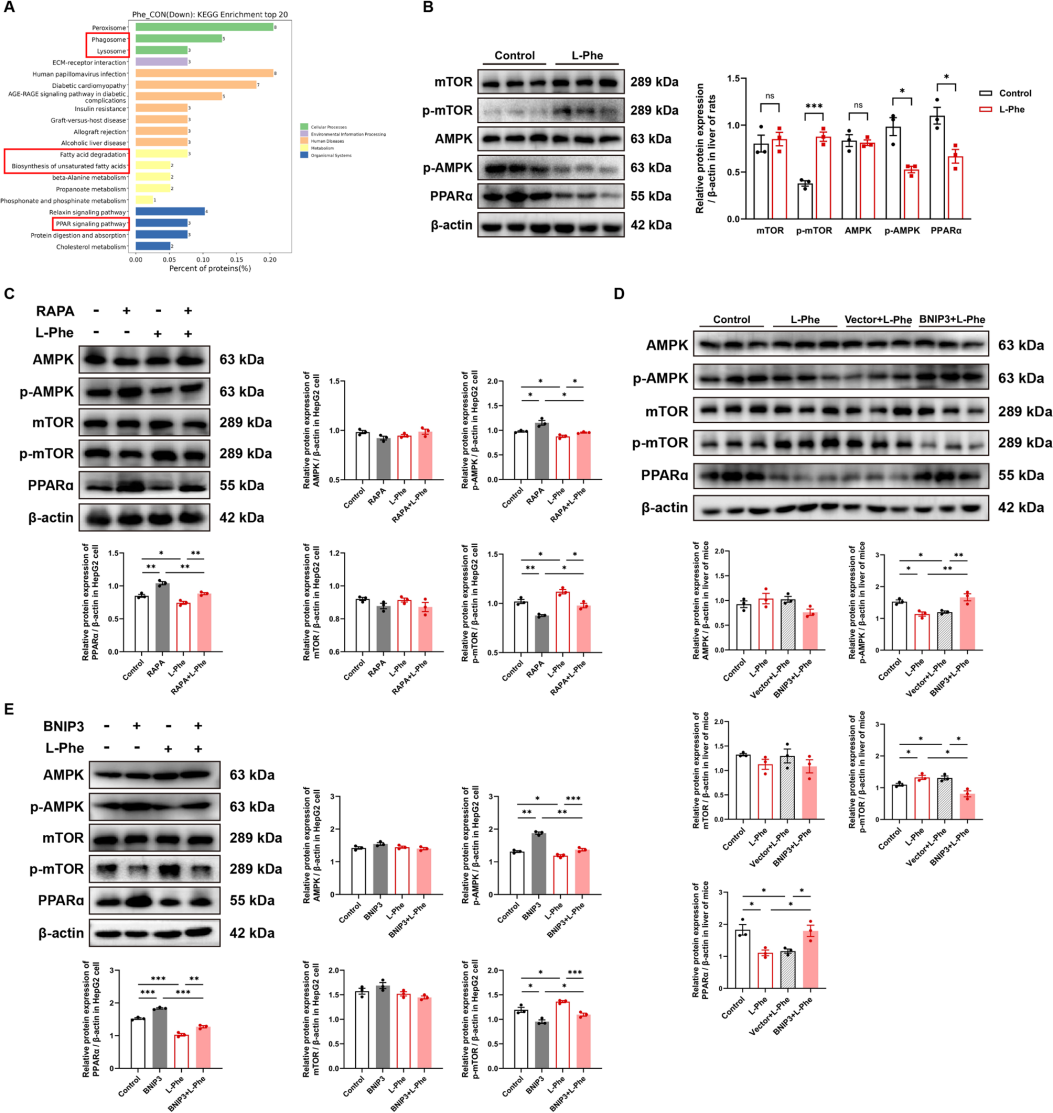
L-Phe regulates the BNIP3-mediated PPARα and AMPK/mTOR signalling pathways to promote hepatic steatosis. **A** Distribution of differentially expressed proteins at KEGG Level 3 for the livers of rats (*n* = 4 per group). The x-axis represents the ratio (%) of differentially expressed proteins annotated to each level 3 metabolic pathway compared to the total number of differentially expressed proteins annotated to all KEGG pathways. The y-axis shows the names of the Level 3 pathways. **B** Western blot analysis (left) and quantification (right) of the expression of proteins in the PPARα and AMPK/mTOR pathways in the livers of rats (*n* = 3 per group). **C** Western blot analysis and quantification of the expression of proteins in the PPARα and AMPK/mTOR pathways in HepG2 cells treated with rapamycin and/or L-Phe (*n* = 3 per group). **D** Western blot analysis and quantification of the expression of proteins in the PPARα and AMPK/mTOR pathways in the livers of mice. **E** Western blot analysis and quantification of PPARα and AMPK/mTOR pathway protein expression in HepG2 cells overexpressing BNIP3 and/or treated with L-Phe (*n* = 3 per group). The data are presented as the means ± SEMs. Two-tailed Student’s t test was used for two-group comparisons, and one-way ANOVA with Tukey post hoc test was used for multigroup comparisons. *p < 0.05, **p < 0.01, ***p < 0.001, ns, not significant

Since high L-Phe levels were found to cause hepatic lipid deposition and disorders of fatty acid metabolism in the liver, we mainly focused on the signalling pathways that might be involved in MASLD reported in recent studies (Diniz et al. 2021; Shen et al. 2023). KEGG pathway enrichment analysis of the differentially expressed proteins in the rats revealed that the phagosome, lysosome, and PPARα pathways were significantly downregulated with L-Phe exposure (Fig. 7A). In addition, the AMPK/mTOR pathway is a key signalling pathway that regulates fatty acid synthesis and mitophagy. Adenosine monophosphate-activated protein kinase (AMPK) activated by phosphorylation maintains mitochondrial homeostasis and lipid homeostasis, which increases fat catabolic processes while decreasing anabolic effects. A previous study reported that BNIP3 deficiency reduces the activation of mTOR and the nuclear translocation of SREBF1 (Mesquita et al. 2020). We then examined the changes in the protein levels of PPARα and the AMPK/mTOR pathway components and found that PPARα and the phosphorylation of AMPK (Thr127) were decreased and the phosphorylation of mTOR (Ser2448) was increased in the L-Phe groups in vivo and in vitro compared with those in the control groups (Fig. 7B-E and Figure S6A-S6F, *p* < 0.05). No significant changes were observed in total AMPK or mTOR protein levels. RAPA or BNIP3 overexpression caused marked activation of PPARα and p‐AMPK (Thr127) and inhibition of p‐mTOR (Ser2448) (Fig. 7C and D, *p* < 0.05). After treatment with L-Phe, these significant effects were alleviated. The levels of PPARα and p‐AMPK (Thr127) decreased, and the level of p‐mTOR (Ser2448) increased in the RAPA + L-Phe and BNIP3 + L-Phe groups compared with the RAPA and BNIP3 groups, respectively (Fig. 7C and E, *p* < 0.05). Moreover, compared to those in the L-Phe group, the levels of PPARα and p‐AMPK (Thr127) were still increased, and the expression of p‐mTOR (Ser2448) was inhibited in the RAPA + L-Phe and BNIP3 + L-Phe groups (Fig. 7C and E, *p* < 0.05). This finding was consistent with that in the livers of the mice (Fig. 7D, *p* < 0.05). Together, these data indicate that L-Phe regulates the PPARα and AMPK/mTOR signalling pathways by inhibiting BNIP3-mediated mitophagy.

## Discussion

**Fig. 8:**
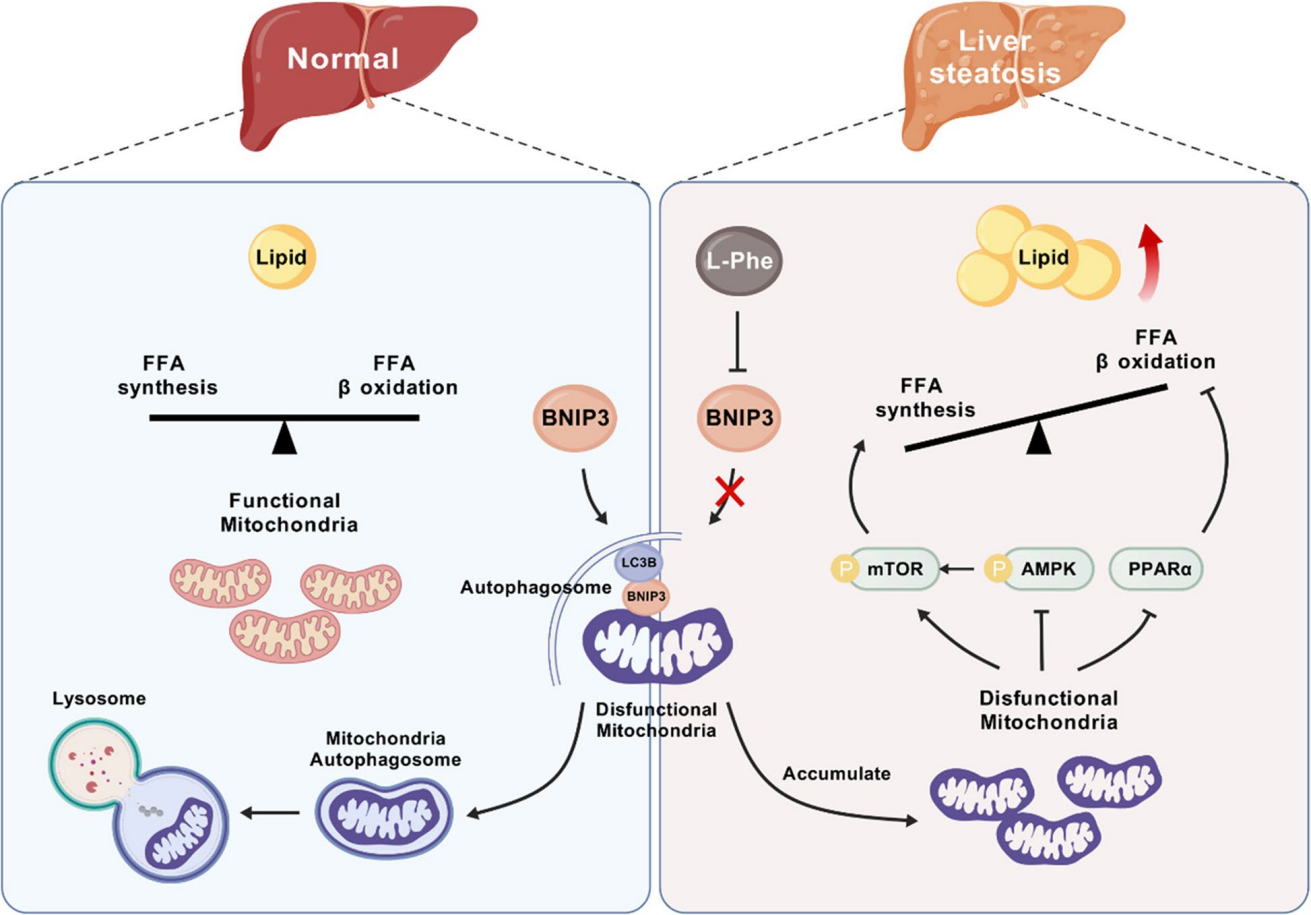
The proposed mechanism by which L-Phe promotes liver steatosis. L-Phe reduces BNIP3 expression, thereby inhibiting BNIP3 recruitment of LC3B to form autophagosomes and initiate mitophagy to clear dysfunctional mitochondria. The accumulation of dysfunctional mitochondria promotes fatty acid synthesis by inhibiting AMPK and activating mTOR while inhibiting fatty acid β-oxidation through the suppression of the PPARα signalling pathway, resulting in lipid droplet deposition in the liver and incident liver steatosis

Previous evidence has shown significant changes in the levels of several circulating amino acids during the onset and progression of MASLD (Gaggini et al. 2018). While abnormal liver function under pathological conditions can lead to fluctuations in L-Phe levels (Masoodi et al. 2021), it remains unclear whether changes in L-Phe levels also impact or mediate liver functions. In this study, data from two distinct populations consistently demonstrated a positive association between L-Phe levels and hepatic lipid metabolism disorders and increased odds of MASLD. In rats, supplementation with L-Phe led to increased lipid accumulation and decreased mitophagy in the liver. We further provide novel evidence suggesting that L-Phe exacerbates hepatic lipid metabolism disorders and promotes liver steatosis potentially through the inhibition of mitophagy (Fig. 8).

Phe is an essential amino acid primarily obtained through dietary intake and is typically present as L-Phe in the body (Alamshah et al. 2017). Hyperphenylalaninemia, which is closely associated with phenylketonuria, is generally characterized by an L-Phe level above 1200 μmol/L (Lichter-Konecki and Vockley 2019). However, the normal range for L-Phe in humans remains uncertain, with one study suggesting a typical range of L-Phe of approximately 48.7 to 85.4 μmol/L (Wang et al. 2022). According to this study, the concentration of L-Phe in the UK Biobank fell within this relatively normal range, while it appeared to be slightly elevated in the SPECT-China population. These differences may be attributed to variations in population characteristics and the methods used for detection. Furthermore, previous studies have shown that diets containing either 0.1% or 1% L-Phe effectively increase serum L-Phe levels in C57BL/6 J mice (Zhou et al. 2022). To establish a mild phenylketonuria model in PAH^enu2^ mice, L-Phe was administered in water at a concentration of 2 g/L (0.2%) (Pascucci et al. 2013). Therefore, for the current study, a mild model was established using drinking water supplemented with 2 g/L L-Phe. The exposure level is approximately four times that of humans, based on an average daily intake of 3.4 g of L-Phe in adults (Blachier et al. 2021).

Several metabolic effects of L-Phe in the body have been studied. For example, L-Phe can exert antihypertensive effects by promoting the synthesis of tetrahydrobiopterin and reducing the production of superoxide, thus protecting blood vessel and kidney function (Heikal et al. 2018). Evidence of whether L-Phe reduces food intake; stimulates the release of CCK, PYY, or GLP-1; and is related to insulin resistance seems to vary across species of experiments and levels of L-Phe intake (Zhou et al. 2022; Alamshah et al. 2017; Fitzgerald et al. 2020; Landgraf et al. 1974). Moreover, R. gnavus can synergize with a HFD to promote hepatic steatosis, possibly potentially through the phenylalanine pathway (Wu et al. 2023). In this study, we found that 2 g/L L-Phe ingested through drinking water did not increase fasting blood glucose or insulin resistance in vivo. However, elevated levels of L-Phe were associated with worsened lipid metabolism in the liver and increased odds of MASLD. Furthermore, we detected a decrease in fatty acid oxidation, consistent with previous results linking the dysregulation of phenylalanine catabolism with lipid accumulation in the context of parenteral nutrition-associated liver disease (Jiang et al. 2023). At the same time, we also observed that L-Phe treatment exacerbated liver steatosis by upregulating fatty acid synthesis, a phenomenon not previously documented. Further research is necessary to validate our findings.

Compared to other amino acids, L-Phe exhibits several distinctive features in MASLD pathogenesis. First, as the primary metabolite of aspartame, the levels of L-Phe increase with consumption of sugar-free beverages, creating a unique diet-environment interaction. Second, L-Phe was the exclusive precursor for tyrosine and catecholamines, enabling neuroendocrine modulation of hepatic lipid metabolism. Third, metabolomic studies consistently identify elevated phenylalanine pathway metabolites in early MASLD, with hepatic-specific metabolic processing suggesting its utility as an early biomarker (Hu et al. 2021; Tan et al. 2024). These characteristics collectively position L-Phe uniquely within the amino acid spectrum in MASLD development.

Our research also revealed a novel role of L-Phe in hepatic lipid metabolism through its impact on mitophagy, showing that L-Phe promotes liver steatosis by inhibiting mitophagy. John Lemasters first coined the term “mitophagy” to describe the selective autophagy of depolarized mitochondria entering acidic lysosomal compartments in hepatocytes (Lemasters et al. 1998). Mitochondria are pivotal for cellular lipid handling and maintain hepatic lipid homeostasis by regulating mitochondrial turnover and function. Dysfunction of this gene can lead to an imbalance in lipid synthesis, oxidation, and storage within hepatocytes (Lu et al. 2023; Wang et al. 2023). Under normal conditions, mitophagy ensures the removal of damaged or dysfunctional mitochondria, thereby preventing the accumulation of reactive oxygen species and toxic lipid intermediates. However, inhibition of mitophagy disrupts this protective mechanism, resulting in the persistence of dysfunctional mitochondria. The accumulation of dysfunctional mitochondria in hepatocytes leads to impaired lipid metabolism characterized by increased lipogenesis and reduced β-oxidation of fatty acids (Ma et al. 2020). Recently, the PINK1-Parkin-mediated mitophagy pathway has been widely studied and shown to be involved in the development of MASLD (Zhou et al. 2019). However, we found no significant differences in the expression of PINK1 or Parkin in this study. Various mitophagy mechanisms, including PINK1/Parkin-mediated, BNIP3/NIX-mediated, FUNDC1-mediated, and AMPK-mediated mitophagy, are activated in response to different stress conditions (Choubey et al. 2021; Zhu et al. 2023). The mechanism by which L-Phe promotes liver steatosis is through the inhibition of BNIP3-mediated mitophagy, as shown in our findings. The results from TEM reveal a spectrum of mitophagosome morphologies, which underscores the dynamic and complex nature of mitophagy. While the variability observed may appear inconsistent, it is in fact consistent with the diverse stages of mitochondrial engulfment and degradation.

BNIP3 is a mitochondrial proapoptotic protein in the BCL-2 superfamily that is localized to the outer mitochondrial membrane and plays an important role in regulating mitochondrial mass and preserving mitochondrial integrity (Zhang and Ney 2009). The BNIP3 protein can directly bind to LC3 through its BH3 domain and induce mitophagy via a nonclassical pathway (Lu et al. 2023). The absence of BNIP3 has been associated with chronic liver damage and metabolic disorders. BNIP3 knockout mice exhibit increased lipid synthesis due to reduced AMPK activity and elevated expression of lipogenic genes. During fasting, hepatic BNIP3 levels are notably elevated in these mice, correlating with increased oxidation in liver (Glick et al. 2012). Given the acknowledged role of BNIP3-mediated mitophagy in liver protection, we investigated whether L-Phe regulates BNIP3-mediated mitophagy in the context of liver steatosis. Our results showed that overexpression of BNIP3 resulted in a decrease in the MMP, increased colocalization of mitochondria and lysosomes, and an increase in the proportion of mitophagosomes. With further treatment with L-Phe, this significantly activated mitophagy is inhibited, and the phenotype of fatty acid disorders in the liver is exacerbated. Based on these findings, it is inferred that liver steatosis caused by L-Phe could be related to BNIP3-mediated mitophagy. However, we cannot confirm whether L-Phe can also affect mitophagy or mitochondrial function through other pathways or regulate fatty acid metabolism through potential metabolic regulatory pathways, which deserves further exploration.

The AMPK pathway is involved in disrupting the equilibrium of redox status between lipogenesis and lipid oxidation (Shen et al. 2019). Activation of AMPK results in the phosphorylation and subsequent inactivation of ACC, preventing the conversion of ACC to malonyl-CoA for fatty acid synthesis and inhibiting hepatic lipid accumulation (Savage et al. 2006). Subsequently, the reduction in malonyl-CoA levels positively regulates the expression of the rate-limiting enzyme CPT1α, thereby promoting mitochondrial lipid oxidation. Moreover, PPARα can control mitochondrial fatty acid β-oxidation through the activation of AMPK to increase fatty acid uptake (Zhang et al. 2023). In the present study, we suspected that the AMPK/mTOR and PPARα pathways are involved in L-Phe-induced liver steatosis. Treatment with RAPA activated the AMPK/mTOR and PPARα signalling pathways, consistent with findings that CCCP directly disrupted the electrochemical gradient of the mitochondrial inner membrane and activated mitophagy (Santarelli et al. 2020). Further exposure to L-Phe inhibited RAPA-induced mitophagy and the AMPK/mTOR and PPARα pathways. Evidence suggests that downregulation of mitophagy can lead to the accumulation of nuclear receptor corepressor 1, which inhibits PPARα activity and leads to impaired lipid oxidative decomposition (Saito et al. 2019). However, previous studies have focused primarily on mTOR-mediated mitophagy and have not explored the bidirectional regulatory relationship between mTOR and mitophagy (Lu et al. 2020; Sharma et al. 2020). Given that alterations in the AMPK/mTOR and PPARα pathways are due to mitophagy inhibition after L-Phe exposure, it is valuable to confirm and investigate the moderating relationships among these pathways in detail in the future.

Several limitations of this study should be considered. First, the precise molecular pathways through which L-Phe regulates BNIP3 remain unclear, representing a critical focus for future investigation. Second, clinical data on mitophagy in human subjects are scarce, precluding direct comparisons with our animal model findings. Third, while animal models provide valuable insights, inherent differences in physiology, metabolism, and disease progression between rodents and humans limit the translational applicability of these results. Fourth, using HepG2 cells, selected for their practicality and established utility in liver steatosis and mitophagy research (Pang et al. 2018; Amorim et al. 2022), cannot fully replicate the complexity of in vivo systems. We acknowledge that primary hepatocytes would offer a more physiologically relevant platform, though their use was constrained by technical challenges for a stable model.

## Conclusions

In summary, we discovered a novel function of L-Phe in the body: downregulating BNIP3-mediated mitophagy and subsequently promoting liver steatosis. This inhibition leads to increased hepatic lipid accumulation through enhanced fatty acid synthesis and reduced fatty acid β-oxidation. These findings provide new insights for the early detection of liver steatosis based on L-Phe levels and suggest potential nutritional intervention strategies for preventing MASLD. However, further research is needed to elucidate the mechanisms by which L-Phe regulates BNIP3.

## Supplementary Information

Supplementary Material 1

## Data Availability

No datasets were generated or analysed during the current study.

## Abbreviations

AAV8: Adeno-associated virus serotype-8
ACAT1: Acetyl-CoA acetyltransferase 1
ACC1: Acetyl‐CoA carboxylase 1
ACOX1: Acyl-CoA oxidase 1
ALT: Aminotransferase
ANOVA: Analysis of variance tests
AST: Aspartate aminotransferase
BMI: Body mass index
CI: Confidence interval
CPT1α: Carnitine palmitoyl transferase 1α
DGAT2: Diacylglycerol O-acyltransferase 2
FASN: Fatty acid synthase
FLI: Fatty liver index
GGT: γ-Glutamyl transferase alanine
GO: Gene ontology
HDL-C: High-density lipoprotein cholesterol
H&E: Hematoxylin and eosin
IHC: Immunohistochemistry
IPGTT: Intraperitoneal glucose tolerance tests
IPITT: Intraperitoneal insulin tolerance tests
KEGG: Kyoto Encyclopedia of Genes and Genomes
LDL-C: Low-density lipoprotein cholesterol
L-Phe: L-Phenylalanine
MASLD: Metabolic dysfunction-associated steatotic liver disease
MMP: Mitochondrial membrane potential
OR: Odds ratio
RAPA: Rapamycin
SPECT-China: Survey on the Prevalence in East China for Metabolic Diseases and Risk Factors
SREBF1: Sterol regulatory element-binding transcription factor 1
TC: Total cholesterol
TEM: Transmission electronic microscopy
TG: Triglyceride
WC: Waist circumference
