# Supplementary material for: L-Phenylalanine promotes liver steatosis by inhibiting BNIP3-mediated mitophagy
Source: Mol Med. 2025 Jun 30;31:250. doi: 10.1186/s10020-025-01303-5 (PMC12207805; doi:10.1186/s10020-025-01303-5)
Supplement: Supplementary file 1 — Supplementary Material 1 [file 10020_2025_1303_MOESM1_ESM.docx]

**Supplementary Materials**

**L-Phenylalanine promotes** **liver steatosis by inhibiting** **BNIP3-mediated mitophagy**

**Table S1.** Definition of MASLD in the UK Biobank and SPECT-Chian populations.

**Table S2.** List of primers used for qPCR.

**Table S3**. List of antibodies used for western blotting.

**Table S4.** Characteristics of participants in the UK Biobank according to the quartiles of L-phenylalanine.

**Table S5.** Characteristics of participants in SPECT-China according to the quartiles of L-phenylalanine.

**Table S6.** Associations between the levels of L-phenylalanine and the odds of MASLD in the UK Biobank and SPECT-China.

**Figure S1**. Flowchart of participants in the UK Biobank and SPECT-China.

**Figure S2.** L-Phe exposure causes weight gain and promotes liver inflammation and adipocyte hypertrophy but does not alter glucose metabolism.

**Figure S3**. L-Phe exposure did not promote apoptosis in the liver of rats and HepG2 cells.

**Figure S4**. Levels of fatty acid metabolism and mitophagy in HepG2 cells exposed to different concentrations of L-Phe.

**Figure S5**. L-Phe exposure causes increased expression of BNIP3 and higher fat mass.

**Figure S6.** Expression levels of AMPK/mTOR and PPARα signalling pathways in HepG2 cells exposed to different concentrations of L-Phe.

**Table S1.** Definition of MASLD in the UK Biobank and SPECT-Chian populations.

|  | UK Biobank | SPECT-China |
| --- | --- | --- |
| Hepatic steatosis | Fatty liver index (FLI) ≥ 60: The FLI is a non-invasive assessment of fatty liver in the general population and was used in a previous study (1). The FLI algorithm included waist circumference (WC), body mass index (BMI), TG, and γ-glutamyl transferase alanine (GGT) as follows:  FLI=e**(0.953*log(TG)+0.139*BMI+0.718*log(GGT)+0.053*WC-15.745)/(1+e**(0.953*log(TG)+0.139*BMI+0.718*log(GGT)+0.053*WC-15.745))×100. | Abdominal ultrasonographic examination (Mindray M7, Minneapolis, Shenzhen, China):  Stronger echoes in the hepatic parenchyma than in the right kidney parenchyma, distal attenuation, vessel blurring, and narrowing of the lumen of the hepatic veins (2). |
| Overweight/obesity | BMI ≥25 kg/m^2^ | BMI ≥24 kg/m^2^ |
| Diabetes | Had self-reported or hospital diagnoses of diabetes (category ID in UK Biobank 1712), were taking antihyperglycemic medications or using insulin, or glycosylated hemoglobin A1c (HbA1c) >6.5% (48 mmol/mol). | Had self-reported or doctor-diagnosed diabetes, were taking antihyperglycemic medications or using insulin, fasting blood glucose ≥ 7.0 mmol/L or HbA1c >6.5% (48 mmol/mol). |
| Metabolic dysregulation | At least two of the following at-risk criteria*:  • Waist circumference ≥102/88 cm in men and women  • Blood pressure ≥130/85 mmHg or specific drug treatment  • Plasma triglycerides ≥150 mg/dl (≥1.70 mmol/L) or specific drug treatment  • Plasma HDL-cholesterol <40 mg/dl (<1.0 mmol/L) for men and <50 mg/dl (<1.3 mmol/L) for women or specific drug treatment.  • Prediabetes (HbA1c 5.7% to 6.4% [39 to 47 mmol/mol])  • Plasma high-sensitivity C-reactive protein level >2 mg/L | At least two of the following at-risk criteria**:  • Waist circumference≥90/80 cm in men and women  • Blood pressure ≥130/85 mmHg or specific drug treatment  • Plasma triglycerides ≥150 mg/dl (≥1.70 mmol/L) or specific drug treatment  • Plasma HDL-cholesterol <40 mg/dl (<1.0 mmol/L) for men and <50 mg/dl (<1.3 mmol/L) for women or specific drug treatment.  • Prediabetes (fasting glucose levels 100 to 125 mg/dl [5.6 to 6.9 mmol/L] or HbA1c 5.7% to 6.4% [39 to 47 mmol/mol])  • Homeostasis model assessment of insulin resistance score (HOMA-IR) ≥ 2.5 |

The fasting blood was not collected at baseline for the UK Biobank study; therefore, data of fasting glucose and HOMA-IR index were not available. Data for high-sensitivity C-reactive protein level in the SPECT-China was not available.

References

1. Zhang H*, et al.* (2022) Non-alcoholic fatty liver disease, sleep behaviors, and incident type 2 diabetes. *J Gastroenterol Hepatol*.

2. Wang N*, et al.* (2018) Vitamin D and Nonalcoholic Fatty Liver Disease: Bi-directional Mendelian Randomization Analysis. *EBioMedicine* **28:** 187-193.

**Table S2.** List of primers used for qPCR.

| Gene | Species | Forward Primer | Reverse Primer |
| --- | --- | --- | --- |
| ACC1 | Rat | GGGAACATCCCCACGCTAAA | GCGCATGGAATGGCAGTAAG |
| FASN | Rat | CACTGGTACACTTTCCCGCT | AGCCTGAGCTTGTCCCTAGA |
| SREBF1 | Rat | CTGTCGTCTACCATAAGCTGCAC | ATAGCATCTCCTGCACACTCAGC |
| DGAT2 | Rat | TGCCAGAACCGTGGTGAAAC | TGGTGGGCCTTAAATCGACC |
| ACOX1 | Rat | AGTCTGAAATCAAGCAAAGC | CATTAATTCGAAGGTAGGTCTC |
| CPT1α | Rat | CCTACCACGGCTGGATGTTT | TACAACATGGGCTTCCGACC |
| ACAT1 | Rat | AGCAAGAGTTCCCACCCACT | TCGAGAAAAACCAGA GGAGGT |
| LPIN1 | Rat | CCACAGAGGGTGGGTCTAGG | GACGTGGCTAGGTACATGGG |
| TNFα | Rat | GACCCTCACACTCAGATCATCTTCT | CCTCCACTTGGTGGTTTGCT |
| IL-1β | Rat | CACCTCTCAAGCAGAGCACAG | GGGTTCCATGGTGAAGTCAAC |
| MCP1 | Rat | GGCCTGTTGTTCACAGTTGCT | GGCCTGTTGTTCACAGTTGCT |
| iNOS | Rat | CCAACCTGCAGGTCTTCGATG | GTCGATGCACAACTGGGTGAAC |
| SOD2 | Rat | TTCAGCCTGCACTGAAG | GTCACGCTTGATAGCCTC |
| CRT | Rat | ATGGCTTTTGACCCAAGCAA | CGGCCCTGAAGCTTTTTGT |
| GPX1 | Rat | CAGTCCACCGTGTATGCCTT | CAGTCCACCGTGTATGCCTT |
| NOX4 | Rat | ACATCCACCAGATGTTGGGC | ACATCCACCAGATGTTGGGC |
| ATG4b | Rat | GGCACTTAGGTCGAGATTGGAG | GGCAGGTCTGGGTTCTCAAC |
| ATG7 | Rat | GTGGCTCCAGGAGATTCCAC | CTCCTCGTCACTCATGTCCC |
| ATG5 | Rat | GGTGACTGGACTTACGGTGG | CATCCAGAGCTGCTTGTGGT |
| Becn1 | Rat | GAATGGAGGGGTCTAAGGCG | CTTCCTCCTGGCTCTCTCCT |
| mt-ND1 | Rat | GCAGGACCATTCGCCCTATT | AAAACGGGGGTAGGATGCTC |
| mt-CO1 | Rat | AGCCGGGGTGTCTTCTATCT | AGCCGGGGTGTCTTCTATCT |
| β-actin | Rat | CCCGCGAGTACAACCTTCTT | AACACAGCCTGGATGGCTAC |

**Table S3**. List of antibodies used for western blotting.

| Antibody | Dilution | Source | Catalogue number |
| --- | --- | --- | --- |
| LAT1 | 1:1000 | HUABIO | HA723086 |
| FASN | 1:1000 | Cell Signaling | 3180 |
| ACC1 | 1:1000 | Cell Signaling | 3676 |
| p-ACC1 | 1:1000 | Cell Signaling | 3661 |
| SREBF1 | 1:1000 | Proteintech | 14088-1-AP |
| CPT1α | 1:1000 | Abcam | ab234111 |
| ACOX1 | 1:1000 | Abcam | ab184032 |
| Cleaved Caspase 3 | 1:1000 | Proteintech | 25128-1-AP |
| PPARα | 1:1000 | Abcam | ab126285 |
| AMPK | 1:1000 | Cell Signaling | 2532 |
| p-AMPK | 1:1000 | Cell Signaling | 2535 |
| mTOR | 1:1000 | Cell Signaling | 2972 |
| p-mTOR | 1:1000 | Cell Signaling | 2971 |
| BNIP3 | 1:1000 | Abcam | ab109362 |
| LC3B | 1:1000 | Abcam | ab192890 |
| P62 | 1:1000 | Abcam | ab109012 |
| PARKIN | 1:1000 | Proteintech | 23274-1-AP |
| PINK1 | 1:500 | Proteintech | 66674-1-Ig |
| FUNDC1 | 1:500 | Proteintech | 28519-1-AP |
| β-actin | 1:5000 | Proteintech | 66009-1-Ig |
| Goat anti-Rabbit | 1:5000 | Proteintech | SA00001-1 |
| Goat anti-Mouse | 1:5000 | Proteintech | SA00001-2 |

**Table S4.** Characteristics of participants in the UK Biobank according to the quartiles of L-phenylalanine.

|  | L-Phenylalanine, μmol/L | | | |  |
| --- | --- | --- | --- | --- | --- |
|  | Quartile 1 | Quartile 2 | Quartile 3 | Quartile 4 | *P* for trend |
|  | (<40.1) | (40.1-46.3) | (46.3-53.4) | (>53.4) |  |
| Number | 45,970 | 45,957 | 45,958 | 45,957 |  |
| Age, years | 55.9±8.2 | 56.3±8.2 | 56.6±8.2 | 56.9±8.1 | <0.001 |
| Men, % | 18,809 (40.9) | 20,755 (45.2) | 22,263 (48.4) | 22,705 (49.4) | <0.001 |
| White, % | 42,968 (93.5) | 43,043 (93.7) | 43,087 (93.7) | 42,974 (93.5) | 0.678 |
| Townsend index | -1.2±3.1 | -1.3±3.1 | -1.3±3.1 | -1.2±3.1 | <0.001 |
| University or college degree, % | 14,263 (31.0) | 14,018 (30.5) | 13,635 (29.7) | 13,510 (29.4) | <0.001 |
| Smoking, % | 4,910 (10.7) | 4,512 (9.8) | 4,168 (9.1) | 4,030 (8.8) | <0.001 |
| Ideal physical activity，% | 20,171 (43.9) | 19,993 (43.5) | 19,805 (43.1) | 19,286 (42.0) | <0.001 |
| WC, cm | 87.3±12.9 | 89.6±13.2 | 91.5±13.5 | 92.8±14.1 | <0.001 |
| BMI, kg/m^2^ | 26.6±4.6 | 27.3±4.7 | 27.9±5.0 | 28.3±5.2 | <0.001 |
| SBP, mmHg | 139.0±19.8 | 139.3±19.7 | 139.4±19.5 | 138.9±19.2 | <0.001 |
| DBP, mmHg | 81.8±10.0 | 82.0±10.1 | 82.0±10.1 | 81.1±10.1 | <0.001 |
| TC, mmol/L | 5.7±1.1 | 5.7±1.1 | 5.6±1.1 | 5.5±1.1 | <0.001 |
| TG, mmol/L | 1.6±0.9 | 1.7±1.0 | 1.8±1.0 | 2.0±1.1 | <0.001 |
| HDL-C, mmol/L | 1.5±0.4 | 1.4±0.4 | 1.4±0.4 | 1.3±0.3 | <0.001 |
| LDL-C, mmol/L | 3.6±0.9 | 3.6±0.9 | 3.5±0.9 | 3.5±0.9 | <0.001 |
| ALT, U/L | 21.9±12.7 | 22.9±13.1 | 23.8±13.7 | 24.5±14.8 | <0.001 |
| AST, U/L | 25.4±9.3 | 25.8±9.4 | 26.1±10.8 | 26.5±10.4 | <0.001 |
| GGT, U/L | 23.6 (17.1, 36.0) | 25.0 (17.9, 38.0) | 26.2 (18.6, 40.1) | 26.8 (18.9, 40.9) | <0.001 |
| FLI≥60, % | 12,961 (28.2) | 16,195 (35.2) | 19,202 (41.8) | 21,605 (47.0) | <0.001 |
| Diabetes, % | 1,991 (4.3) | 2,305 (5.0) | 2,845 (6.2) | 3,455 (7.5) | <0.001 |
| Overweight/obesity, % | 27,393 (59.6) | 30,198 (65.7) | 32,392 (70.5) | 33,517 (72.9) | <0.001 |
| Metabolic disorders, % | 14,020 (30.5) | 12,423 (27.0) | 10,909 (23.7) | 9,523 (20.7) | <0.001 |
| MASLD, % | 12,703 (27.6) | 15,898 (34.6) | 18,905 (41.1) | 21,246 (46.2) | <0.001 |

The data are presented as the mean ± standard deviation (SD) or median (interquartile range) for continuous variables and as a percentage (%) for categorical variables. WC, waist circumference; BMI, body mass index; SBP, systolic blood pressure; DBP, diastolic blood pressure; TC, triglyceride; TG, triglyceride; HDL-C, high-density lipoprotein cholesterol; LDL-C, low-density lipoprotein cholesterol; ALT, alanine transaminase; AST, aspartate transaminase; GGT, γ-glutamyl transferase; FLI, fatty liver index; MASLD, metabolic dysfunction-associated steatotic liver disease. Linear regression was used to compare continuous variables across L-Phe quartiles, while chi-square tests were used for categorical variables.

**Table S5.** Characteristics of participants in SPECT-China according to the quartiles of L-phenylalanine.

|  | L-Phenylalanine, μmol/L | | | |  |
| --- | --- | --- | --- | --- | --- |
|  | Quartile 1 | Quartile 2 | Quartile 3 | Quartile 4 | *P* for trend |
|  | (<111.8) | (111.8-121.6) | (121.6-132.4) | (>132.4) |  |
| Number | 38 | 39 | 39 | 39 |  |
| Age, years | 52.0±1.9 | 52.3±1.7 | 52.2±1.7 | 53.1±1.4 | 0.006 |
| Men, % | 15 (39.5) | 14 (35.9) | 11 (28.2) | 16 (41.0) | 0.935 |
| Urban, % | 11 (28.9) | 9 (23.1) | 6 (15.4) | 5 (12.8) | 0.053 |
| High school education or above, % | 10 (26.3) | 9 (23.1) | 10 (25.6) | 8 (20.5) | 0.63 |
| Smoking, % | 7 (18.4) | 8 (20.5) | 7 (17.9) | 10 (20.6) | 0.513 |
| Ideal physical activity，% | 6 (15.8) | 3 (7.7) | 9 (23.1) | 7 (17.9) | 0.406 |
| WC, cm | 78.2±9.9 | 79.0±8.4 | 78.4±7.5 | 84.7±7.9 | 0.109 |
| BMI, kg/m^2^ | 23.7±3.5 | 23.9±3.1 | 24.8±2.8 | 25.5±2.9 | 0.010 |
| SBP, mmHg | 127.3±18.8 | 128.3±14.3 | 132.1±20.2 | 133.6±19.2 | 0.085 |
| DBP, mmHg | 76.8±12.7 | 80.0±15.5 | 79.5±13.0 | 81.9±13.5 | 0.146 |
| TC, mmol/L | 5.0±1.3 | 5.3±0.8 | 5.2±0.8 | 5.1±0.9 | 0.631 |
| TG, mmol/L | 1.5±1.2 | 1.6±1.0 | 1.5±1.1 | 1.8±1.3 | 0.028 |
| HDL-C, mmol/L | 1.4±0.3 | 1.5±0.3 | 1.4±0.3 | 1.4±0.4 | 0.462 |
| LDL-C, mmol/L | 2.8±0.9 | 3.0±0.6 | 3.0±0.6 | 2.9±0.5 | 0.556 |
| ALT, U/L | 19.0±8.1 | 19.7±8.7 | 19.0±8.3 | 22.2±9.0 | 0.038 |
| AST, U/L | 21.2±4.9 | 23.2±5.2 | 22.6±4.7 | 24.5±6.4 | 0.017 |
| Hepatic steatosis, % | 11 (28.9) | 16 (41.0) | 18 (46.2) | 20 (51.3) | 0.045 |
| Diabetes, % | 3 (7.9) | 2 (5.1) | 4 (10.3) | 5 (12.8) | 0.335 |
| Overweight/obesity, % | 23 (60.5) | 22 (56.4) | 27 (69.2) | 30 (76.9) | 0.070 |
| Metabolic disorders, % | 19 (50.0) | 21 (53.8) | 21 (53.8) | 23 (59.0) | 0.454 |
| MASLD, % | 9 (23.7) | 14 (35.9) | 17 (43.6) | 20 (51.3) | 0.011 |

The data are presented as the means ± standard deviations (SDs) for continuous variables and percentages (%) for categorical variables. WC, waist circumference; BMI, body mass index; SBP, systolic blood pressure; DBP, diastolic blood pressure; TC, triglycerides; TG, triglyceride HDL-C, high-density lipoprotein cholesterol; LDL-C, low-density lipoprotein cholesterol; ALT, alanine transaminase; AST, aspartate transaminase; MASLD, metabolic dysfunction-associated steatotic liver disease. Linear regression was used to compare continuous variables across L-Phe quartiles, while chi-square tests were used for categorical variables.

**Table S6.** Associations between the levels of L-phenylalanine and the odds of MASLD in the UK Biobank and SPECT-China.

| L-Phe | Case | Number | Model 1 | Model 2 | Model 3 |
| --- | --- | --- | --- | --- | --- |
| **UK Biobank** |  |  |  |  |  |
| Q1 | 12,703 | 45,970 | 1.00 (reference) | 1.00 (reference) | 1.00 (reference) |
| Q2 | 15,898 | 45,957 | 1.39 (1.35-1.42) | 1.34 (1.30-1.38) | 1.35 (1.31-1.39) |
| Q3 | 18,905 | 45,958 | 1.83 (1.78-1.88) | 1.73 (1.69-1.78) | 1.75 (1.70-1.80) |
| Q4 | 21,246 | 45,957 | 2.25 (2.19-2.31) | 2.13 (2.07-2.19) | 2.14 (2.08-2.20) |
| *P* for trend |  |  | <0.001 | <0.001 | <0.001 |
| Per 1 SD increase | |  | 1.37 (1.35-1.38) | 1.34 (1.33-1.36) | 1.34 (1.33-1.36) |
| *P* value |  |  | <0.001 | <0.001 | <0.001 |
| **SPECT-China** | |  |  |  |  |
| Q1 | 9 | 38 | 1.00 (reference) | 1.00 (reference) | 1.00 (reference) |
| Q2 | 14 | 39 | 1.80 (0.67-4.87) | 1.83 (0.67-4.99) | 2.07 (0.73-5.87) |
| Q3 | 17 | 39 | 2.49 (0.94-6.63) | 2.67 (0.98-7.23) | 3.22 (1.13-9.19) |
| Q4 | 20 | 39 | 3.39 (1.28-9.01) | 3.18 (1.16-8.69) | 3.98 (1.38-11.45) |
| *P* for trend |  |  | 0.011 | 0.018 | 0.007 |
| Per 1 SD increase | |  | 1.70 (1.16-2.48) | 1.64 (1.11-2.42) | 1.77 (1.16-2.69) |
| *P* value |  |  | 0.006 | 0.013 | 0.008 |

Logistic regression was used, and the data are presented as odds ratios (95% confidence intervals). Model 1 was crude; Model 2 was adjusted for age and sex; Model 3 for the UK Biobank was adjusted for age, sex, race, Townsend deprivation index, education, current smoking, physical activity, and diabetes; Model 3 for SPECT-China was adjusted for age, sex, urban, education, current smoking, physical activity, and diabetes. MASLD, metabolic dysfunction-associated steatotic liver disease; SD, standard deviation.


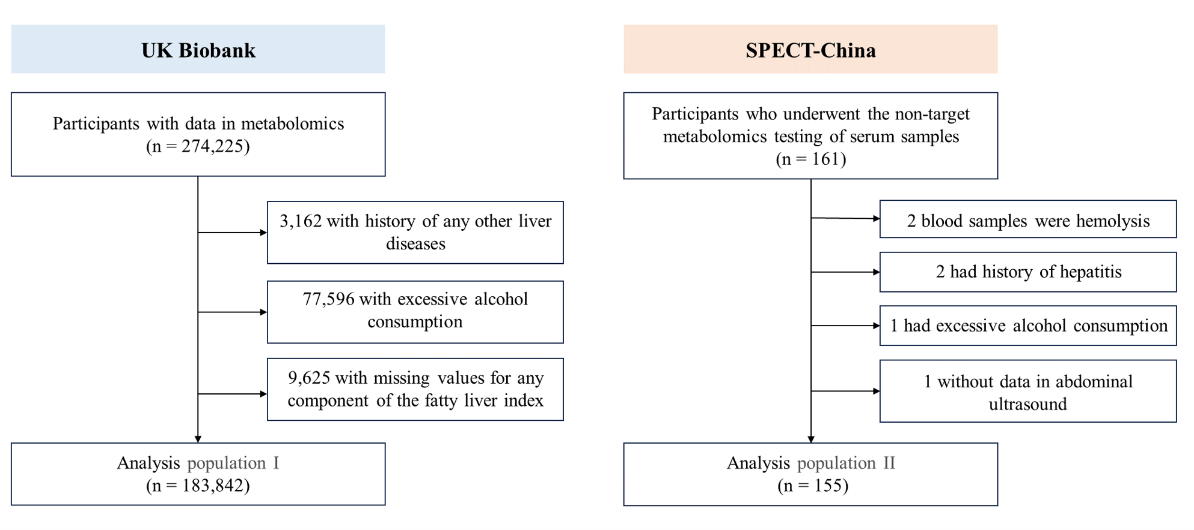


**Figure S1.** Flowchart of participants in the UK Biobank and SPECT-China.


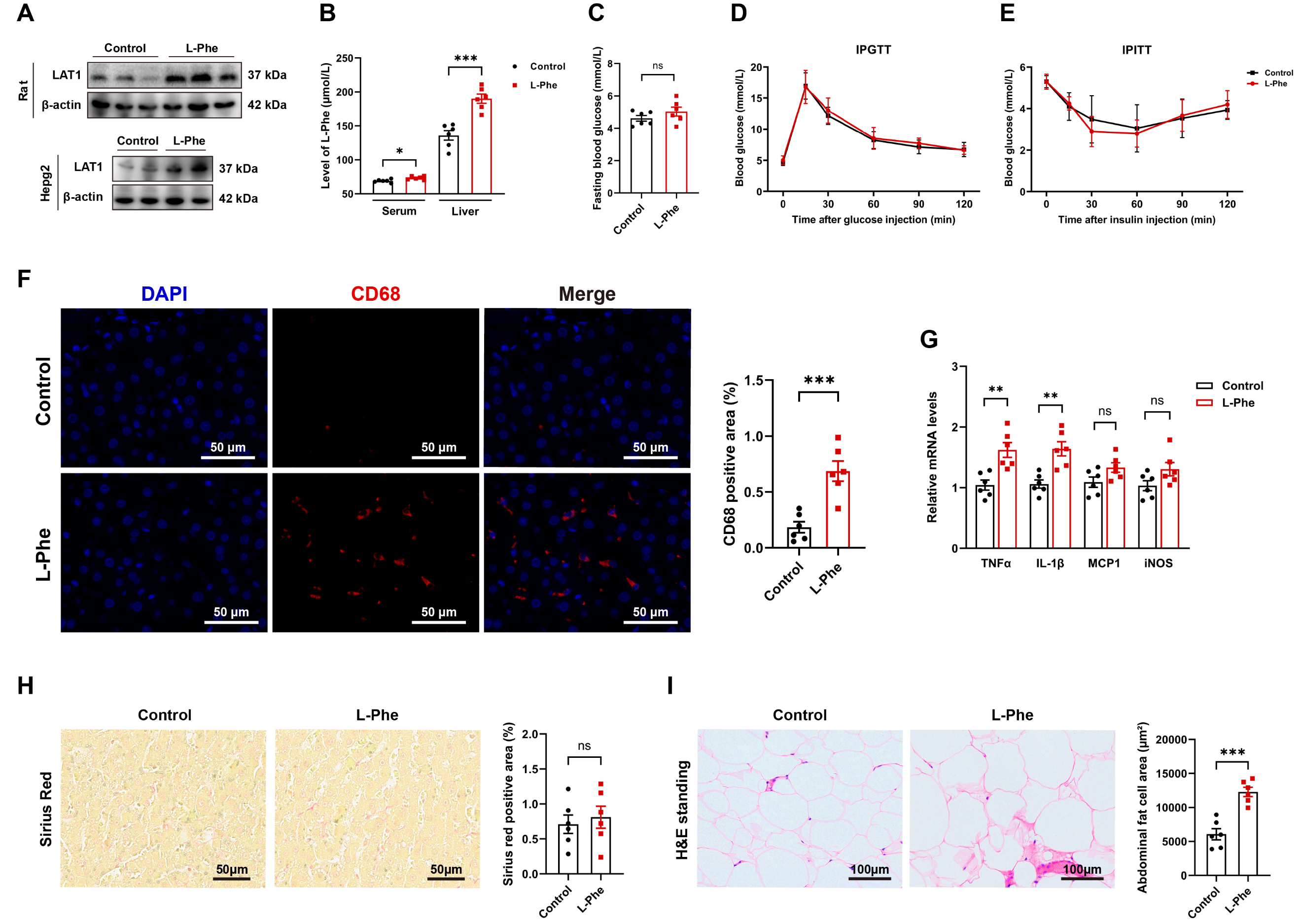


**Figure S2.** L-Phe exposure causes weight gain and promotes liver inflammation and adipocyte hypertrophy but does not alter glucose metabolism.

(A) The protein expression levels of LAT1 in vivo and in vitro models wither exposure to L-Phe.

(B) Levels of L-Phe in serum and liver for the two groups of rats.

(C) Fasting blood glucose levels of rats in week 28 for the two groups of rats.

(D) Glucose tolerance test measuring serum glucose in fasted rats after injection with 1 g/kg glucose. IPGTT, Intraperitoneal Glucose Tolerance Test.

(E) Insulin tolerance test measuring serum glucose in fasted rats after injection with 1 U/kg insulin. IPITT, Intraperitoneal Insulin Tolerance Test.

(F) Immunofluorescence staining (left) and quantification (right) of CD68 (red) and DAPI (blue) in liver sections from rats.

(G) Relative mRNA levels of proinflammatory genes in the livers of the rats.

(H) Sirius Red staining (left) and quantification (right) of livers sections from the two groups of rats.

(I) H&E staining (left) and quantification (right) of visceral adipose tissue sections from the two groups of rats.

The data are presented as the means ± SEMs. Two-tailed Student’s t test was used for two-group comparisons. **p* < 0.05, ***p* < 0.01, ****p* < 0.001, ns, not significant.


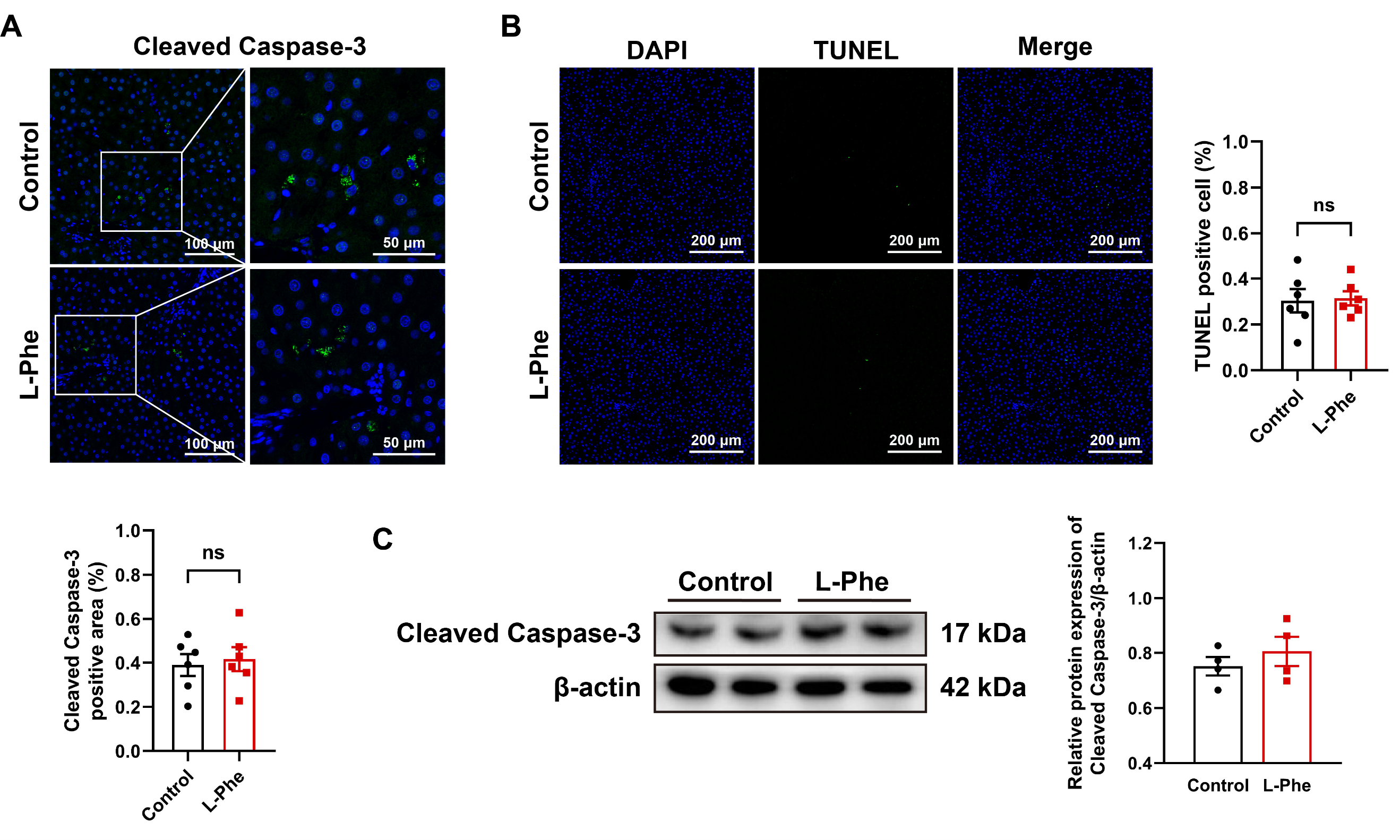


**Figure S3.** L-Phe exposure did not promote apoptosis in the liver of rats and HepG2 cells.

(A) Immunofluorescence for the level of Cleaved Caspase-3 in the livers of rats (green: Cleaved Caspase-3, blue: DAPI).

(B) Representative images (left) and quantification (right) of TUNEL assay in the livers of rats (green: TUNEL-positive cells, blue: DAPI).

(C) The protein expression levels of Cleaved Caspase-3 in vitro models wither exposure to L-Phe.

Two-tailed Student’s t test was used for two-group comparisons. ns, not significant.


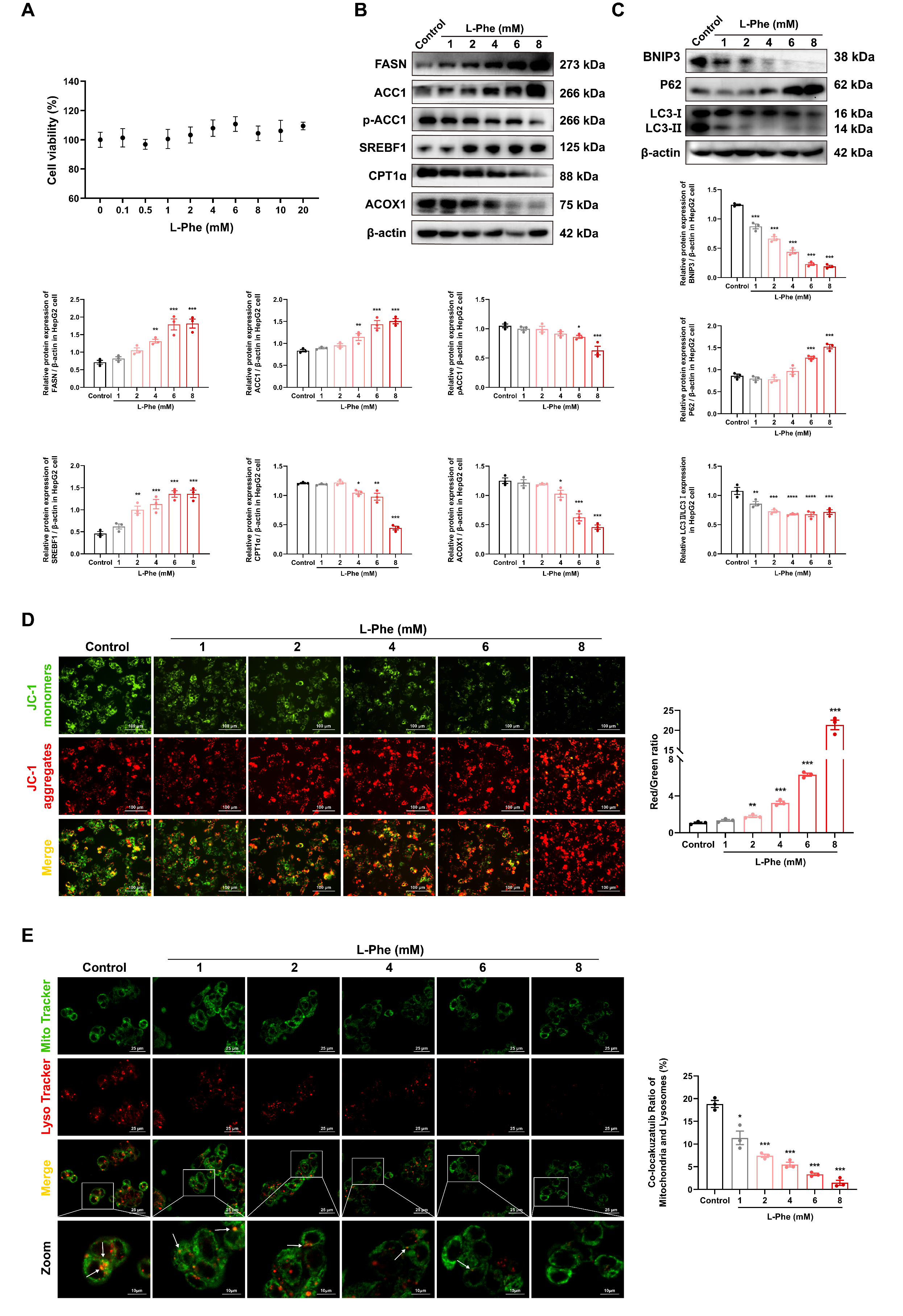


**Figure S4**. Levels of fatty acid metabolism and mitophagy in HepG2 cells exposed to different concentrations of L-Phe.

(A) Effects of L-Phe on HepG2 cells viability assessed by CCK-8 assay.

(B) Western blot analysis (left) and quantification (right) of proteins involved in fatty acid metabolism in HepG2 cells treated with different concentrations of L-Phe.

(C) Western blot analysis (left) and quantification (right) of mitophagy proteins in HepG2 cells treated with different concentrations of L-Phe.

(D) JC-1 staining and quantification of the mitochondrial membrane potential in HepG2 cells. The ratio is the JC-1 aggregate (red) to monomer (green) ratio.

(E) Colocalization of mitochondria (green) and lysosomes (red) labelled with MitoTracker Green and LysoTracker Red (left), respectively, and the percentage of yellow puncta colabelled with MitoTracker Green and LysoTracker Red (right).

The data are presented as the means ± SEMs. One-way ANOVA with Tukey post hoc test was used for multigroup comparisons. **p* < 0.05, ***p* < 0.01, ****p* < 0.001.


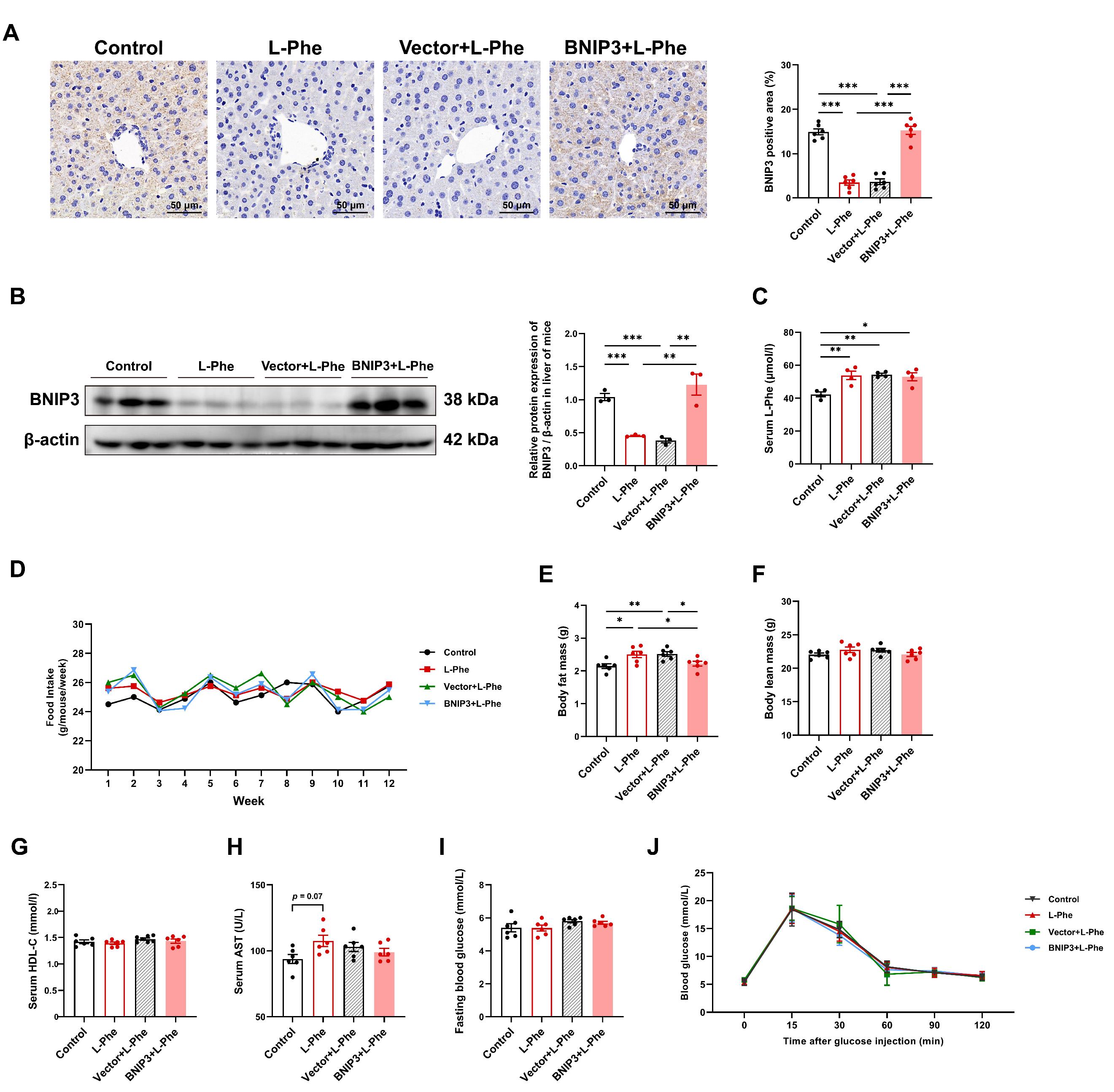


**Figure S5**. L-Phe exposure causes increased expression of BNIP3 and higher fat mass.

(A) Immunohistochemical staining for BNIP3 (left) and quantification (right) of liver sections from the mice.

(B) Western blot analysis (left) and quantification (right) of BNIP3 protein in the livers of mice.

(C) Serum levels of L-Phe in the mice.

(D) Food intake of mice monitored over a 12-week period of L-Phe drinking.

(E and F) Quantification of body fat mass (E) and lean mass (F) in the mice.

(G-I) Serum levels of high-density lipoprotein cholesterol (G), aspartate aminotransferase (H), and fasting blood glucose (I) in the mice.

(J) Glucose tolerance test measuring serum glucose in fasted mice after injection with 1 g/kg glucose.

The data are presented as the means ± SEMs. One-way ANOVA with Tukey post hoc test was used for multigroup comparisons. **p* < 0.05, ***p* < 0.01, ****p* < 0.001.

**
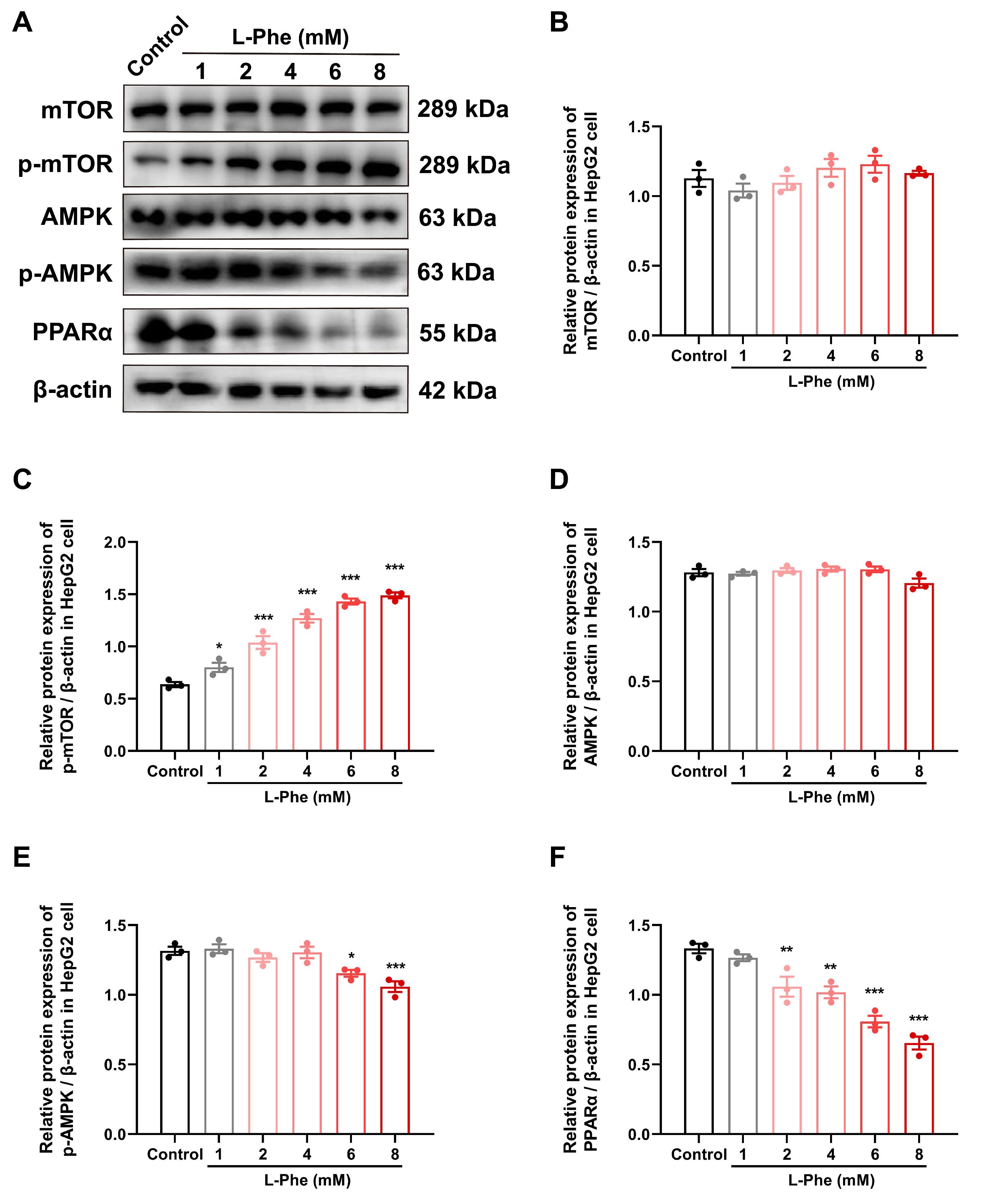
**

**Figure S****6.** Expression levels of AMPK/mTOR and PPARα signalling pathways in HepG2 cells exposed to different concentrations of L-Phe.

(A-F) Western blot analysis and quantification of the expression of proteins in the PPARα and AMPK/mTOR pathways in HepG2 cells treated with different concentrations of L-Phe. The data are presented as the means ± SEMs. One-way ANOVA with Tukey post hoc test was used for multigroup comparisons. The reference group was the control group. **p* < 0.05, ***p* < 0.01, ****p* < 0.001.
